# Prion protein modulates iron transport in the anterior segment: Implications for ocular iron homeostasis and prion transmission

**DOI:** 10.1016/j.exer.2018.05.031

**Published:** 2018-05-31

**Authors:** Ajay Ashok, Shilpita Karmakar, Rajeev Chandel, Ranjana Ravikumar, Stuti Dalai, Qingzhong Kong, Neena Singh

**Affiliations:** Department of Pathology, School of Medicine, Case Western Reserve University, Cleveland, OH, 44106, USA

**Keywords:** Prion protein, Ciliary epithelium, Cornea, Aqueous humor, Iron, β-cleavage

## Abstract

Iron is an essential biometal in the aqueous humor, the principal source of nutrients for the avascular cornea and the lens. Here, we explored whether the ciliary body (CB), the source of aqueous humor, transports iron, and if the prion protein (PrP^C^) facilitates this process as in the outer retina. Using a combination of human, bovine, and mouse eyes as models, we report the expression of iron export proteins ferroportin and ceruloplasmin, and major iron uptake and storage proteins transferrin, transferrin receptor, and ferritin in the ciliary epithelium, indicating active exchange of iron at this site. Ferroportin and transferrin receptor are also expressed in the corneal endothelium. However, the relative expression of iron export and uptake proteins suggests export from the ciliary epithelium and import by corneal endothelium. In addition, abundant expression of PrP^C^, a ferrireductase that facilitates iron transport, is noted in pigmented and non-pigmented epithelium of the CB, posterior pigmented epithelium of the iris, corneal endothelium and epithelium, and lens epithelium. Notably, majority of PrP^C^ in the ciliary epithelium is cleaved at the β-site as in retinal pigment epithelial cells, suggesting a role in iron transport. Most of the PrP^C^ in the cornea, however, is full-length, and susceptible to aggregation by intracerebrally inoculated PrP-scrapie, an infectious conformation of PrP^C^ responsible for human and animal prion disorders. Soluble PrP^C^ is present in the aqueous and vitreous humor, a provocative observation with significant implications. Together, these observations suggest independent cycling of iron in the anterior segment, and a prominent role of PrP^C^ in this process. Aggregation of PrP^C^ in the cornea of PrP-scrapie-infected animals raises the alarming possibility of transmission of animal prions through corneal abrasions.

## 1. Introduction

Iron is an essential catalyst for vital biochemical reactions because of the ease with which it can donate and accept electrons. The same reaction, however, can cause cytotoxicity by Fenton chemistry, requiring tight regulation of iron homeostasis at multiple levels (Ganz, 2013). In the systemic circulation, iron is maintained within safe limits by regulating uptake from the intestine (Ganz, 2013). The eye is protected further from fluctuations in systemic iron by the outer and inner blood-retinal barriers comprised of retinal pigment epithelial cells and capillary endothelial cells respectively, and the blood aqueous barrier formed by epithelial cells of the ciliary body (CB). Although the regulation of iron transport across blood-retinal barriers has been the subject of several recent reports (Asthana et al., 2017; Chaudhary et al., 2018; Sterling et al., 2017), relatively little is known about the exchange of iron, if any, across the blood-aqueous barrier.

The cornea and the lens are avascular, and acquire iron and other nutrients from the aqueous humor (AH) secreted mainly by epithelial cells of the ciliary body (CB) (Bishop et al., 2002). Morphologically, the CB is comprised of capillary-rich stroma enclosed by ciliary processes lined by a double layer of epithelial cells. The inner pigmented layer is continuous with retinal pigment epithelial cells of the outer retina. The outer layer is non-pigmented, faces the AH, and is continuous with neural retina. Non-pigmented epithelial (NPE) cells are polarized and form the functional blood-aqueous barrier. The apical surface of pigmented epithelium (PE) and NPE is juxtaposed, and separated by gap junctions to form a syncytium. This results in the coupling of PE and NPE cells in trans-epithelial transport. The polarized distribution of ion transporters in these cells determines the transport of solutes and water in the desired direction. (Delamere, 2005). Proteome analysis of the AH suggests active synthesis and transport of several proteins by the ciliary epithelium, including proteins involved in iron transport (Bertazolli-Filho et al., 2003, 2006; Chowdhury et al., 2010).

Current understanding of iron cycling in the eye suggests that iron is transported from the vitreous to the lens equatorial junction and the epithelial-fiber interface before export to the AH by lens epithelial cells. An alternate hypothesis suggests that Tf-iron from the vitreous diffuses directly to the AH along a concentration gradient (Garcia-Castineiras, 2010). Based on this information, the only source of iron for the AH is the retina. It is unclear whether the CB, the main source of AH, plays an active role in iron transport although it is believed to synthesize and secrete Tf and the ferroxidases ceruloplasmin (Cp) and hephaestin required for iron export (Bertazolli-Filho et al., 2003; Janssen et al., 2013). Since iron dyshomeostasis in the anterior segment is likely to increase the vulnerability of the cornea and lens to photo-oxidative damage, this is an important unresolved question with significant clinical implications.

Recently, we reported that the prion protein (PrP^C^), the principal protein implicated in the pathogenesis of human and animal prion disorders, is expressed on retinal pigment epithelial cells and facilitates iron uptake by functioning as a ferrireductase partner for divalent metal transporters (Asthana et al., 2017). Since this layer is continuous with pigmented epithelium of the CB, it is plausible that PrP^C^ plays a similar role at this site. Here, we explored this possibility using human, bovine, and mouse eyes as models. Our data support active exchange of iron across the ciliary epithelium and corneal endothelium, and a prominent role of PrP^C^ in this process. Moreover, corneal PrP^C^ in mouse models of chronic wasting disease (CWD) undergoes a conformational change to the aggregated, potentially infectious PrP-scrapie isoform (Kong et al., 2005), suggesting the possibility of prion transmission through the cornea.

## 2. Materials and methods

### 2.1. Animals and ethics statement

All animals were housed in the Animal Resource Center at Case Western Reserve University School of Medicine approved by the Association for Assessment and Accreditation of Laboratory Animal Care International under a 12 h day-night cycle, and provided ad libitum access to food and water. All experimental procedures were reviewed and approved by the IACUC committee in accordance with provisions of the Animal Welfare Act and Guide for the Care and Use and of Laboratory Animals as well as the U.S. Government Principles for the Utilization and Care of Vertebrate Animals Used in Testing, Research, and Training (animal protocol # 2015–0027). The animal resource center at Case Western reserve university is directed by Dr. Durfee, DVM, Diplomate ACLAM, and daily animal care is provided by two full-time veterinarians. The PHS Assurance number A-3145–01 is valid until 04/30/19.

### 2.2. Mouse models

PrP-knock-out (PrP^−/−^) mice were originally obtained from George Carlson and bred on the FVBN/J background for more than 15 generations. Transgenic mice expressing 2× levels of human PrP^C^ on the FVBN/J PrP^−/−^ background and cervid PrP^C^ were generated in our facility (Kong et al., 2005). A combination of male and female mice between 2 and 4 months of age were used for all experiments. All animals were kept under a 12 h day-night cycle and had ad libitum access to food and water.

### 2.3. Human and bovine samples

Human eye globes were acquired from the Minnesota Eye Bank. The globes were either dissected to isolate different parts of the eye or fixed in buffered formalin (1:10) for 24 h before processing for immunohistochemistry. Freshly harvested bovine eyes were collected from a local abattoir and processed as above.

### 2.4. Antibodies and chemicals

The following antibodies were used in this study: Anti-PrP antibodies 3F4 and 8H4 (Signet Laboratories, Dedham, MA), and SAF32 (189720, Cayman chemicals, USA), anti-ferritin (F5012, Sigma Aldrich, USA), anti-TfR (136800, Invitrogen, USA), anti-Tf (GTX2123, GeneTEX, USA), anti-ceruloplasmin (Dako, USA), Mouse IgG (5415, Cell signaling Technology, USA), anti-Fpn (NBP1-21502, Novus biological, USA), anti β-actin (MAB1501, Milipore, USA) and anti GAPDH (GT239, GeneTEX). HRP-conjugated anti-mouse, NA931V and anti-rabbit, NA934V (GE Healthcare, UK). Alexa Fluor-conjugated secondary antibodies (molecular probes), PNGase F (P0704S, NEB, USA). NAP (Non-animal protein) blocking solution (786-190T, G-Biosciences, USA), Hoechst (#33342, Invitrogen, USA), Fluoromount-G (Southern Biotech, USA).

### 2.5. Tissue preparation and immunohistochemistry

Age-matched mice were euthanized, and enucleated eyes were fixed for 24 h in 1:10 buffered formalin. Fixed eyes were paraffin embedded, and thin sections were immunostained as described (Asthana et al., 2017). In brief, sections were rehydrated with xylene followed by decreasing concentrations of ethanol (100%, 90% and 70%), and finally, three washes of 5 min each with 1× PBS. Antigen retrieval was performed by heating the tissue at 97 °C in the presence of 25 mM tris-1 mM EDTA (pH-8.5) for 30 min. Non-specific sites were blocked with 1% BSA for mouse and human tissue, and NAP blocker (G-biosciences, St. Louis MO) for bovine tissue. The tissues were subsequently washed and incubated with respective primary antibody followed by incubation with the corresponding Alexa Fluor conjugated secondary antibodies. The nuclei were stained with Hoechst (#33342, Invitrogen, USA) and the sections were mounted using Fluoromount-G (Southern Biotech, USA). Images were captured with Leica inverted microscope (DMi8).

### 2.6. Isolation of the cornea, ciliary body, and neuroretina

The cornea, CB, and retina were isolated as described (McMenamin, 2000) under a dissecting microscope (LEICA S4E). For bovine and human samples, the separation of different structures was complete. For mouse samples, however, the CB was contaminated with pieces of iris. The neuroretina was isolated effectively by scraping off the tissue gently. All samples were lysed immediately and processed for western blotting.

### 2.7. Preparation of lysates from lens epithelial cells and corneal endothelial cells

Lens epithelial cells eyes were isolated and processed as described (Long et al., 2008). For bovine samples endothelial cells were scraped from freshly harvested corneas and lysed for evaluation. For mouse samples whole corneas were lysed and extracted proteins analyzed. Thus, mouse samples included a mixture of corneal epithelial and endothelial cells.

### 2.8. Infection with CWD

Transgenic mice expressing cervid PrP^C^ (Kong et al., 2005) were injected intracerebrally with 30 μL of brain homogenate from CWD-infected elk. Infected animals were euthanized when clinical signs of disease appeared after ~141 days post inoculation, and the cornea, CB, and retina isolated from enucleated eyes were lysed and processed for differential centrifugation and Western blotting as reported (Baksi et al., 2016). Uninoculated control transgenic mice were analyzed in parallel.

### 2.9. SDS-PAGE and western blotting

Samples were prepared in RIPA lysis buffer (50 mM Tris-Cl pH7.4, 100 mM NaCl, l%NP-40, 0.5% deoxycholate) and were clarified by centrifugation, boiled in reducing gel-loading buffer, and fractionated by SDS-PAGE. Proteins transferred to a PVDF membrane were probed with specific antibodies and developed by chemiluminescence as described (Baksi and Singh, 2017). Quantification of protein bands was performed by densitometry using UN-SCANT-IT (version6.1) software (Silk Scientific) and represented graphically. Dilutions of antibodies used for immunoblotting were 3F4 (1:500), 8H4 (1:500), Ferritin (1:1000), TfR (1:1000), Tf (1:1000), β-actin (1:5000), Gapdh (1:1000), HRP-mouse (1:15,000), HRP-rabbit (1:15000).

### 2.10. Statistical analysis

The data were analyzed using GraphPad Prism5 software (GraphPad software, Inc., La Jolla, CA) and represented as Mean ± SEM. Level of significance was calculated by unpaired *t*-test between control and experimental samples.

## 3. Results

A combination of human, bovine, and mouse eyes were used to increase the validity of our results. Mouse models expressed either no PrP (PrP^−/−^), or 2× human PrP in the PrP^−/−^ background (PrP^+/+^) (Kong et al., 2005). Human and bovine eyes represented diurnal species, and eyes from mice, a nocturnal species, facilitated investigations on the functional significance of PrP^C^ in iron transport. A comparison of iron transport proteins in diurnal and nocturnal species is essential because the former are more susceptible to iron-induced photo-oxidative damage, and expression of certain proteins may vary accordingly (VázQuEz-QuIñoNES and García-Castiñeiras, 2009).

### 3.1. The ciliary body modulates iron homeostasis in the anterior segment

**Fig. 1.**
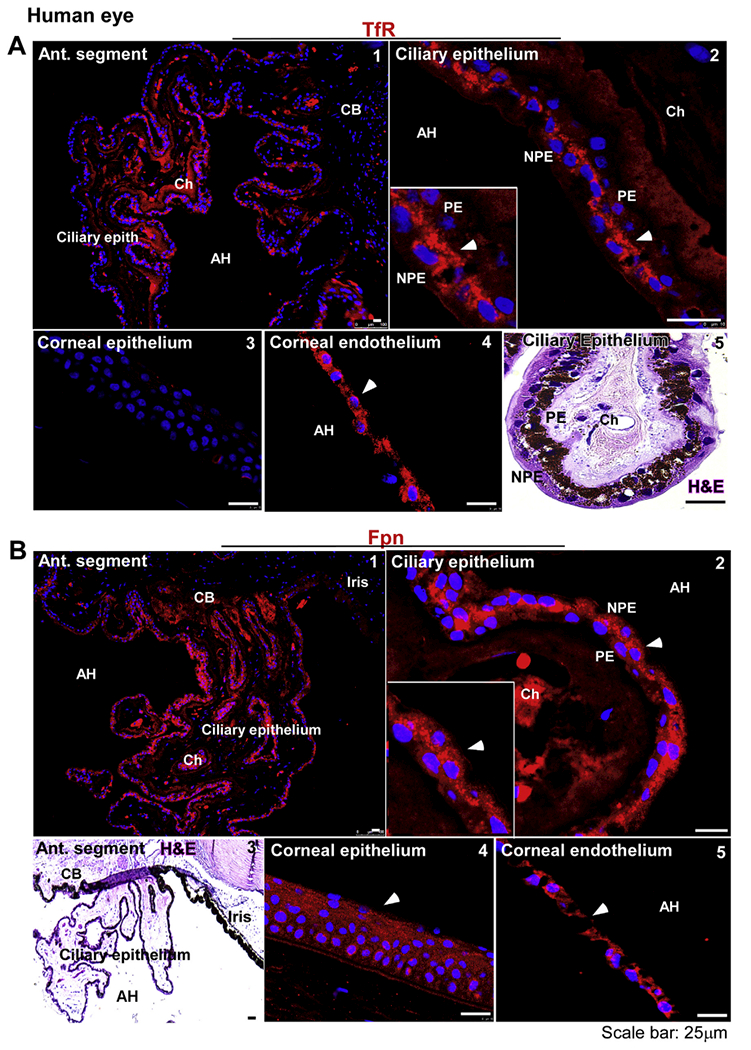
Distribution of TfR and Fpn in the anterior segment of human eye: **(A)** Immunostaining for TfR shows uniform expression on the ciliary epithelium (panel 1). The reactivity for TfR is limited to the apical domain and intracellular vesicles of NPE cells (panel 2). Corneal epithelium does not express the TfR (panel 3). Corneal endothelium, on the other hand, shows strong reactivity for TfR in intracellular vesicles (panel 4). H&E stained section of the ciliary epithelium shows PE and NPE around a stroma rich in choroidal capillaries (panel 5). Scale bar: 25 μm. **(B)** Immunostaining for Fpn shows uniform reactivity on the ciliary epithelium (panel 1). Fpn is expressed mainly in NPE cells (panel 2). H&E stained section shows orientation of the ciliary epithelium with respect to the iris and AH (panel 3). Fpn is expressed on corneal epithelium (panel 4) and endothelium (panel 5). Scale bar: 25 μm.

To evaluate whether the CB contributes to iron cycling in the AS, thin sections of fixed human eye were immunostained with antibodies for TfR and Fpn, principal iron uptake and export proteins. Imaging of ciliary processes in the posterior chamber showed uniform reactivity for TfR on epithelial cells bathed by the AH (Fig. 1 A, panel 1). Higher resolution images revealed apical and cytosolic localization of TfR in NPE cells. (Fig. 1 A, panel 2). Corneal epithelial cells did not show expression of TfR (Fig. 1 A, panel 3), though corneal endothelial cells reacted strongly (Fig. 1 A, panel 4). H&E stained section of the ciliary epithelium highlights its structure and the localization of PE and NPE cells (Fig. 1 A, panel 5).

A similar evaluation for Fpn showed a strong reaction along the ciliary epithelium facing the AH (Fig. 1 B, panel 1). Most of the reactivity for Fpn was distributed intracellularly in NPE cells, though occasionally PE cell also showed some reaction (Fig. 1 B, panel 2). H&E stained section shows the orientation of ciliary epithelium relative to the stroma and the iris (Fig. 1 B, panel 3). Corneal epithelium and endothelium also reacted strongly for Fpn (Fig. 1 B, panels 4 & 5).

**Fig. 2.**
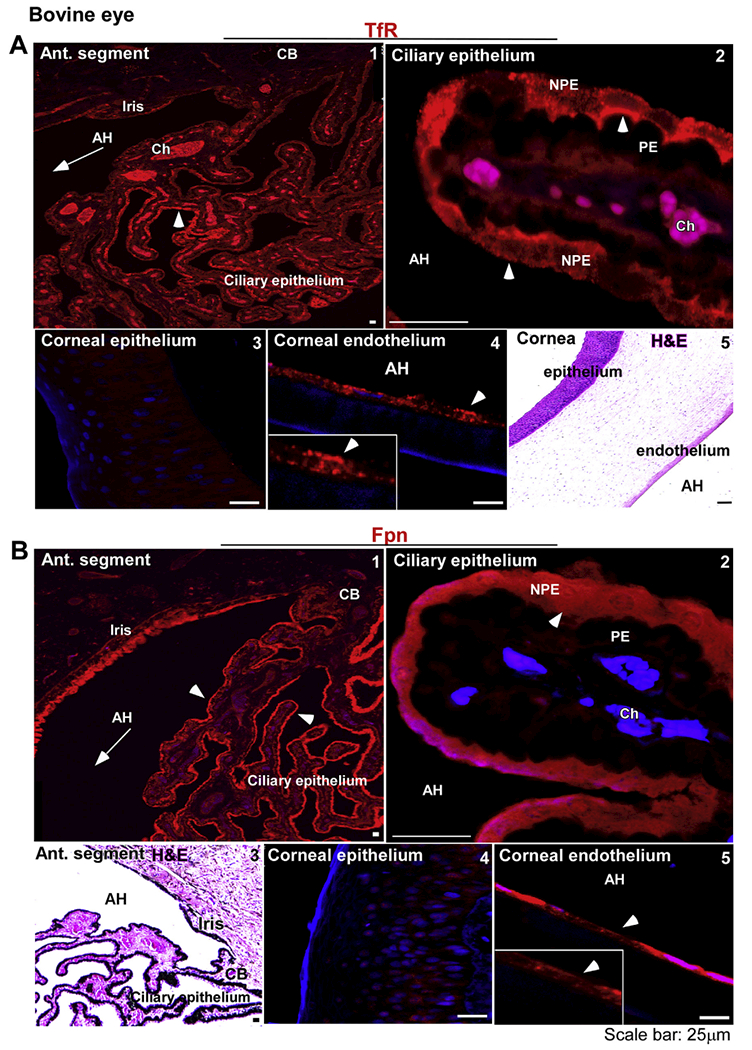
Distribution of TfR and Fpn in the anterior segment of bovine eye: **(A)** The reaction for TfR is evident on the ciliary epithelium facing the AH (panel 1). TfR is localized on the apical membrane and intracellular vesicles of NPE cells as in the human sample (panel 2). The corneal epithelium does not react for TfR (panel 3), while the corneal endothelium shows a distinct reaction on the plasma membrane (panel 4). H&E staining shows multiple layers of corneal epithelium, the stroma, and a single layer of corneal endothelium (panel 5). Scale bar: 25 μm. **(B)** Fpn is expressed on the ciliary epithelium and the iris (panel 1). The expression of Fpn is limited to NPE cells as in the human sample (panel 2). H&E staining shows the orientation of ciliary epithelium with respect to the iris and AH (panel 3). Unlike human samples, the bovine corneal epithelium did not react for Fpn (panel 4). However, corneal endothelium showed strong reactivity for Fpn (panel 5). Scale bar: 25 μm.

The above observations were confirmed in bovine eyes due to their larger size and availability of multiple samples of different ages. As noted in the human sample, the reactivity for TfR was distributed throughout the ciliary epithelium (Fig. 2 A, panel 1). Higher resolution images revealed apical and cytosolic distribution of TfR in NPE cells, though some cells showed a uniform distribution on the basolateral membrane as well (Fig. 2 A, panel 2). Corneal epithelium did not show detectable expression of TfR (Fig. 2 A, panel 3). However, corneal endothelial cells showed strong reactivity on the plasma membrane (Fig. 2 A, panel 4). H&E stained section of the cornea shows the multilayer corneal epithelium and a monolayer of corneal endothelial cells as expected (Fig. 2 A, panel 5).

Evaluation of Fpn expression showed uniform distribution along the ciliary epithelium and posterior pigment epithelium of the iris (Fig. 2 B, panel 1). Further evaluation revealed intracellular localization of Fpn in NPE cells as in the human sample (Fig. 1 B, panel 2). H&E section from a similar region shows the orientation of the ciliary epithelium with respect to the iris (Fig. 1 B, panel 3). Unlike the human sample, reactivity for Fpn was absent in the corneal epithelium (Fig. 2 B, panel 4), though a strong reaction was detected in corneal endothelial cells (Fig. 2 B, panel 5).

**Fig. 3.**
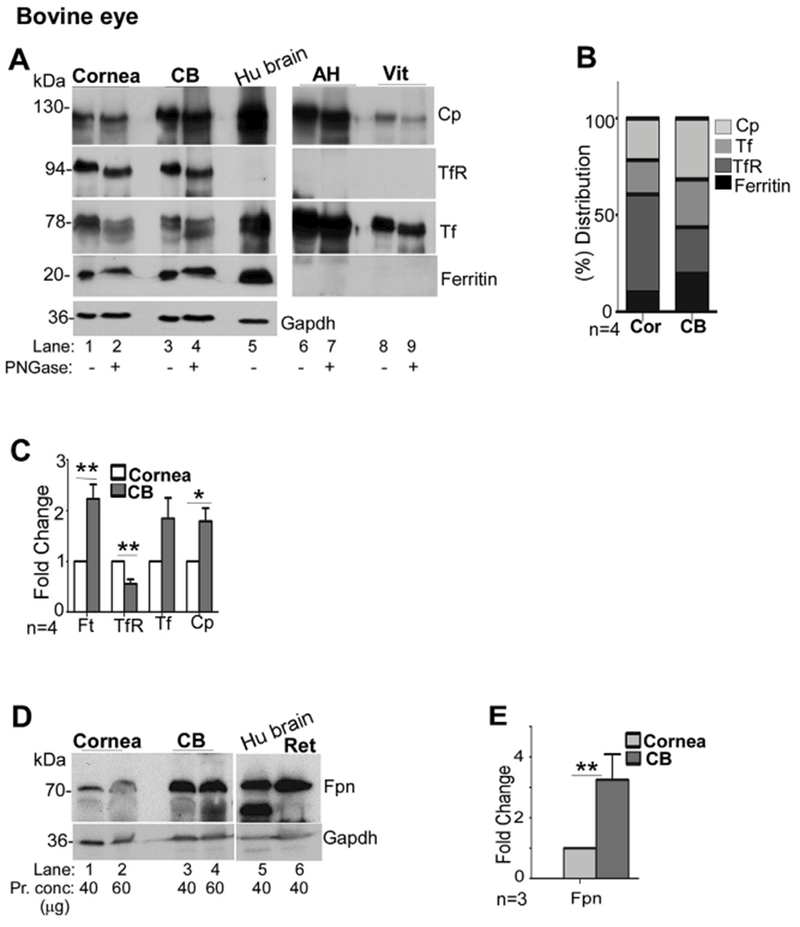
Expression of iron modulating proteins in the cornea and ciliary body. **(A)** Probing of lysates from bovine cornea (Cor) and CB for Cp, TfR, Tf, and ferritin shows the expression of all of these proteins in both samples (lanes 1–4). Deglycosylation results in faster migration of Cp, TfR, and Tf on SDS-PAGE, indicating the presence of glycans (lanes 2 & 4). AH and the vitreous show abundant presence of Cp and Tf, both of which migrate faster upon deglycosylation (lanes 7 & 9). No reactivity for TfR or ferritin is detected in these samples (lanes 6–9). Human brain lysate was processed in parallel as a positive control (lane 5). Gapdh served as a loading control. (Cor: cornea; Ft: ferritin). **(B)** Relative distribution of iron modulating proteins within each tissue shows higher expression of TfR relative to Cp and ferritin in the cornea, and higher levels of Cp relative to the TfR and ferritin in the CB. **(C)** Quantitative comparison of protein expression by densitometry shows significantly higher levels of ferritin and Cp, and lower levels of TfR in the CB relative to the cornea. All values were normalized to Gapdh that provided the loading control. Values represent fold change ± SEM of the indicated n. **(D)** Probing of Western blots of bovine cornea and CB for Fpn revealed increased expression of Fpn (3.2 fold) in CB relative to the cornea (lanes 1–4). Lysates from human brain and bovine retina were analyzed in parallel as controls (lanes 5 & 6). Gapdh served as a loading control. **(E)** Quantification by densitometry shows 3.2 fold higher levels of Fpn in the CB relative to the cornea. Values are mean + SEM of the indicated n. **p < 0.01. The full images of the cropped blots have been provided in the Supplementary Data.

Further confirmation of the above results was obtained by analyzing protein extracts by Western blotting. Accordingly, ten bovine eyes from different animals were dissected, and lysates prepared from the cornea and CB were pooled. Likewise, the AH and the vitreous were pooled. After thorough mixing, equal protein from each sample was subjected to Western blotting prior to or following deglycosylation with PNGase F. Human brain lysate was fractionated in parallel as a positive control (Fig. 3 A).

Probing of transferred proteins for major iron modulating proteins showed a robust reaction for Cp, TfR, Tf, and ferritin in the cornea, CB and human brain (Fig. 3 A, lanes 1–5). The AH and the vitreous showed significant amounts of Cp and Tf, but lacked TfR and ferritin as expected (VázQuEz-QuIñoNES and García-Castiñeiras, 2009) (Fig. 3 A, lanes 6–9). Deglycosylation revealed a detectable change in the migration of Cp, TfR, and Tf in all samples, confirming the presence of glycans. There was no change in the migration of ferritin as expected (Fig. 3 A, lanes 1–4 & 6–9). Evaluation of relative protein expression in corneal and CB lysates revealed TfR as the major protein in the cornea, and Cp and ferritin in the CB (Fig. 3 B). Quantitative comparison by densitometry showed significantly higher expression of ferritin and Cp and lower levels of TfR in the CB relative to the cornea, supporting the above observations (Fig. 3 C). Evaluation of Fpn expression revealed significantly higher levels in the CB relative to the cornea (Fig. 3 D & E).

Together, the above results indicate active export of iron by the CB and import by the corneal endothelium. Since the ciliary epithelium is a continuation of the retina, further studies were focused on whether PrP^C^ facilitates iron transport across these cells as reported for RPE cells in the outer retina (Asthana et al., 2017).

### 3.2. PrP^C^ is expressed in the anterior segment of human, bovine, and mouse eyes

**Fig. 4.**
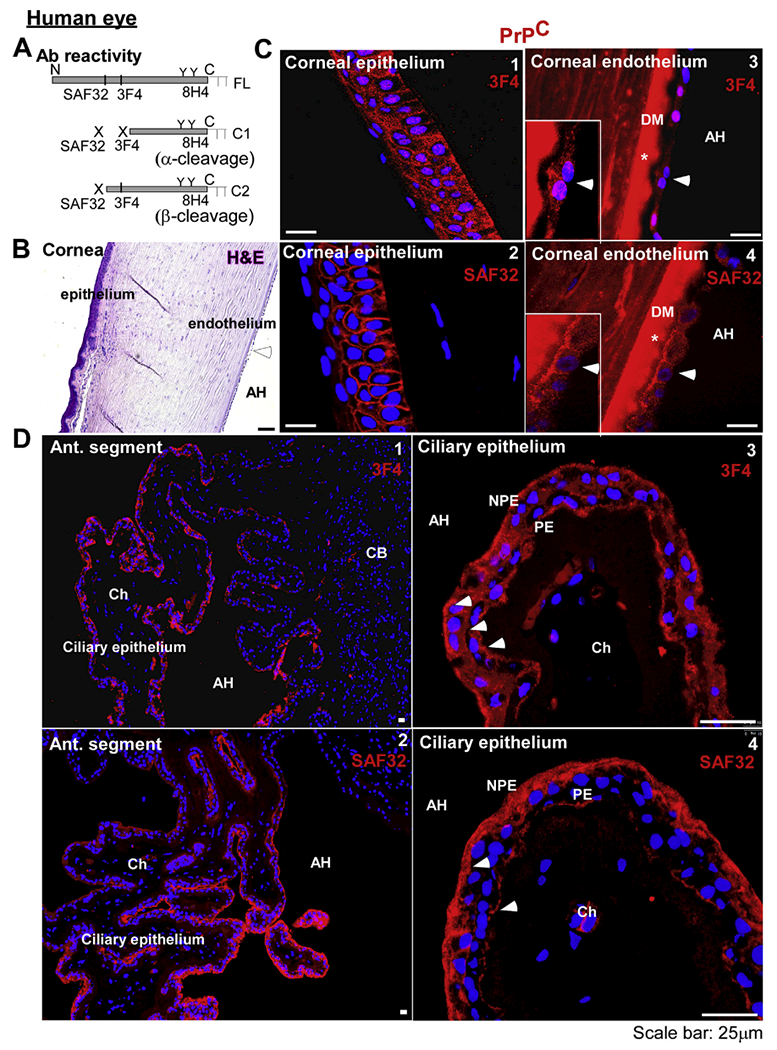
PrP^C^ is expressed widely in the anterior segment of the human eye: **(A)** Schematic representation of full length (FL), α-cleaved (Cl), and β-cleaved (C2) forms of PrP^C^ and antibody reactivity. **(B)** H&E staining of the human cornea shows several layers of epithelium, stromal fibers, and a monolayer of endothelial cells. **(C)** Immunoreaction of corneal sections with 3F4 and SAF32 shows surface and intracellular reactivity in epithelial (panels 1 & 2) and endothelial cells (panels 3 & 4). *Reactivity with Descemet’s membrane (DM) is non-specific (negative control in supplementary data, Fig. 1). Scale bar: 25 μm. **(D)** Immunoreaction of the ciliary body with 3F4 and SAF32 shows a positive reaction in the ciliary epithelium (panels 1 &2). Reactivity with 3F4 is prominent on the basolateral membrane of PE and NPE, and intracellular vesicles in NPE cells (panel 3). Reaction with SAF32 is more prominent on the basolateral surface and intracellular vesicles of NPE cells (panel 4). Scale bar: 25 μm.

To evaluate whether PrP^C^ is expressed in the CB and the cornea, sections of human eye were stained with H&E or immunoreacted with PrP-specific antibodies SAF32, 3F4, and 8H4 to identify species-specific and proteolytically processed forms of PrP^C^ (Fig. 4 A). SAF32 and 8H4 react with human, bovine, and mouse PrP^C^ while 3F4 reacts only with human PrP^C^. Since the epitopes for these antibodies differ, reactivity with a specific antibody combined with molecular mass on SDS-PAGE provides a convenient method to differentiate between full-length, α-cleaved, and β-cleaved forms of PrP^C^. Thus, the epitope for 3F4 between residues 109–112 of human sequence is disrupted by α-cleavage at residues 111/112, but spared by β-cleavage near residue 90 (McDonald et al., 2014; Watt and Hooper, 2005). SAF32 reacts with residues 63–94 in the octapeptide repeat region, and thus reacts with only full-length PrP^C^, not α- and β-cleaved C-terminal fragments. 8H4, on the other hand, reacts with C-terminal residues 145–180, and reacts with full-length α-cleaved (Cl), and β-cleaved (C2) forms of PrP^C^ (Fig. 4 A).

H&E staining of the cornea showed multiple layers of non-keratinized, stratified squamous epithelial cells on the anterior surface, collagen fibers in the stroma, and a single layer of corneal endothelial cells resting on the Descemet’s membrane as expected (Fig. 4 B). Immunoreaction with 3F4 and SAF32 revealed intracellular and cell-surface reactivity for PrP^C^ in the corneal epithelium and endothelium, indicating the presence of full-length and possibly β-cleaved form of PrP^C^ (Fig. 4 C, panels 1–4). The reaction with DM is non-specific (negative control staining in Fig. 1-Supplementary data).

Immunoreaction of the CB with 3F4 and SAF32 showed widespread expression of PrP^C^ on ciliary epithelial cells (Fig. 4 D, panels 1 & 2). Higher resolution images revealed strong reactivity with 3F4 on the basolateral surface and intracellular vesicles of PE and NPE cells (Fig. 4 D, panel 3). Immunoreaction with SAF32, however, revealed a slightly different pattern. PrP^C^ was distributed mainly on the basolateral surface and intracellular vesicles of NPE cells. PE cells showed a uniform but relatively less reaction on the basolateral membrane (Fig. 4 D, panel 4). Based on the epitopes of these antibodies (Fig. 4 A), these results suggest that most of the PrP^C^ on NPE cells is full-length and cleaved at the β-site in PE cells.

**Fig. 5.**
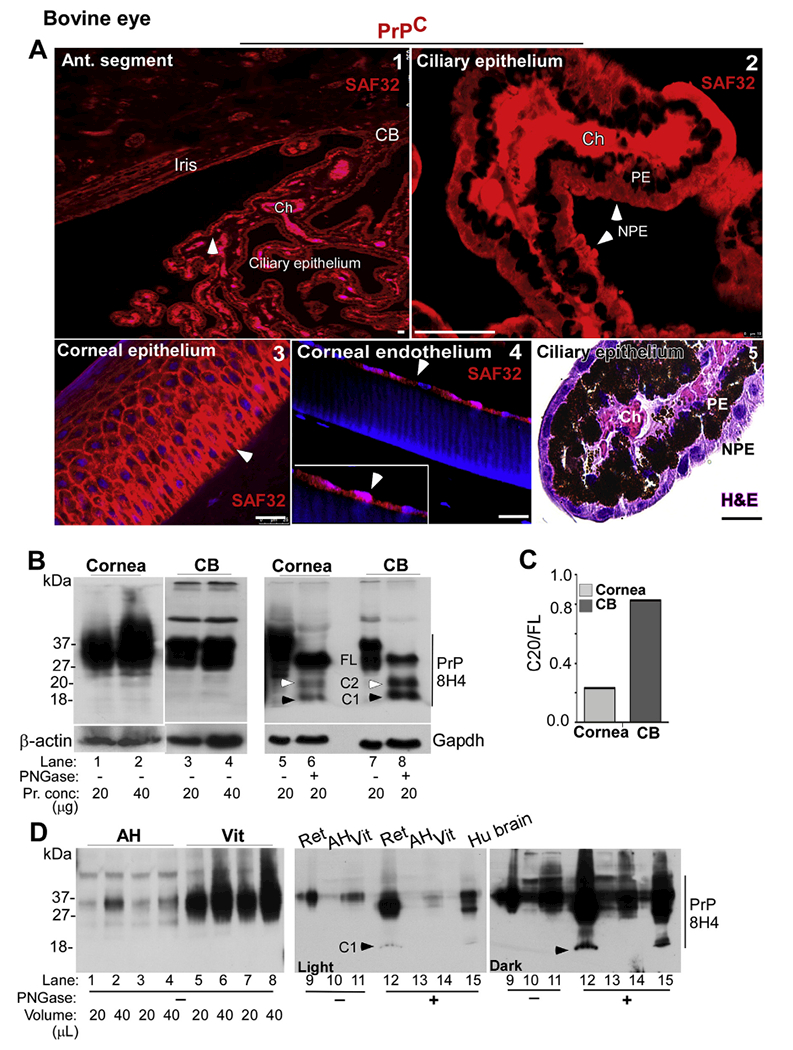
Distribution and processing of PrP^C^ in the anterior segment of bovine eye: **(A)** Immunoreaction of sections from the CB with SAF32 shows a prominent reaction on the ciliary epithelium (panel 1). Reactivity for PrP^C^ is localized to the basolateral membrane and intracellular vesicles of NPE cells (panel 2). Corneal epithelium (panel 3) and endothelium (panel 4) show strong reactivity for PrP^C^ on the plasma membrane and intracellular vesicles. H&E staining shows the clear distinction between PE and NPE cells (panel 5). Scale bar: 25 μm. **(B)** Immunoblotting of corneal and CB lysates with 8H4 shows the expected glycoforms of PrP^C^ (lanes 1–4). Deglycosylation shows mainly FL PrP^C^ in the cornea (lanes 5 & 6). The CB, however, shows α- and β-deaved C1 and C2 fragments in addition to FL PrP^C^ (lanes 5–8). β-actin and Gapdh served as loading controls. **(C)** The ratio of C2 vs. FL in the CB is 3.5 fold more than the cornea. **(D)** Probing of AH and vitreous with 8H4 shows glycosylated forms of PrP^C^ (lanes 1–8). Deglycosylation of AH and vitreous samples and retinal and human brain lysates fractionated in parallel shows the presence of Cl fragment in the retina and brain, but not in the AH and the vitreous (lanes 9–15; lanes 12 & 15 vs. 13 & 14). Full images of cropped blots are included in the Supplementary Data.

Likewise, immunoreaction of bovine samples with SAF32 revealed strong reactivity for PrP^C^ on the ciliary epithelium, localized mostly on the basolateral membrane and intracellular vesicles of NPE cells (Fig. 5 A, panels 1 & 2). Panel 5 is an H&E stained section for better visualization of PE and NPE cells in the ciliary epithelium (Fig. 5 A). Corneal epithelium and endothelium also reacted strongly for PrP^C^ as in the human sample (Fig. 5 A, panels 3 & 4).

To confirm the above observations, pooled lysates from the cornea and CB of 10 bovine eyes were analyzed by Western blotting. Equal protein from each sample was deglycosylated with PNGase, and untreated and deglycosylated samples were fractionated by SDS-PAGE followed by Western blotting (Fig. 5 B). Probing with 8H4 showed the expected full-length glycoforms of PrP^C^ in the cornea and CB (Fig. 5 B, lanes 1–4). However, deglycosylation revealed mostly full-length PrP^C^ in corneal endothelial cells, and equivalent amounts of full-length, β-cleaved, and α-cleaved forms of PrP^C^ in the CB, releasing C-terminal fragments C2 and Cl of 20kDa and 18kDa respectively (Fig. 5 B, lanes 5–8). Quantification of the ratio of C2 vs. full-length PrP^C^ revealed higher levels of C2 in the CB relative to corneal endothelial cells (Fig. 5 C).

To determine the presence of soluble PrP^C^ in the AH and the vitreous, equal volume of each from pooled samples was processed for Western blotting as above. Lysates from bovine retina and human brain were fractionated in parallel as controls (Fig. 5 D). Probing with 8H4 revealed full-length glycosylated forms of PrP^C^ in the AH, and significantly higher levels in the vitreous (Fig. 5 D, lanes 1–8). Deglycosylation prior to Western blotting revealed the presence of α-cleaved Cl fragment of PrP^C^ in retinal and brain lysates, but not in samples from AH and the vitreous (Fig. 5 D, lanes 12 & 15 vs. 13 & 14). It is notable that α-cleavage occurs in an endocytic compartment during recycling of PrP^C^ from the plasma membrane (Chen et al., 1995), and its absence suggests the presence of soluble PrP^C^ in the AH and the vitreous.

### 3.3. PrP^C^ facilitates iron transport across the ciliary epithelium

The expression of PrP^C^ in the ciliary epithelium and corneal endothelium suggested a functional role at these sites. To explore this possibility, subsequent experiments were conducted on mouse models lacking PrP^C^ (PrP^−/−^) or expressing human PrP^C^ (PrP^+/+^) to determine whether PrP^C^ modulates iron transport as reported in RPE cells and other cell types (Asthana et al., 2017) (Haldar et al., 2015; Tripathi et al., 2015).

**Fig. 6.**
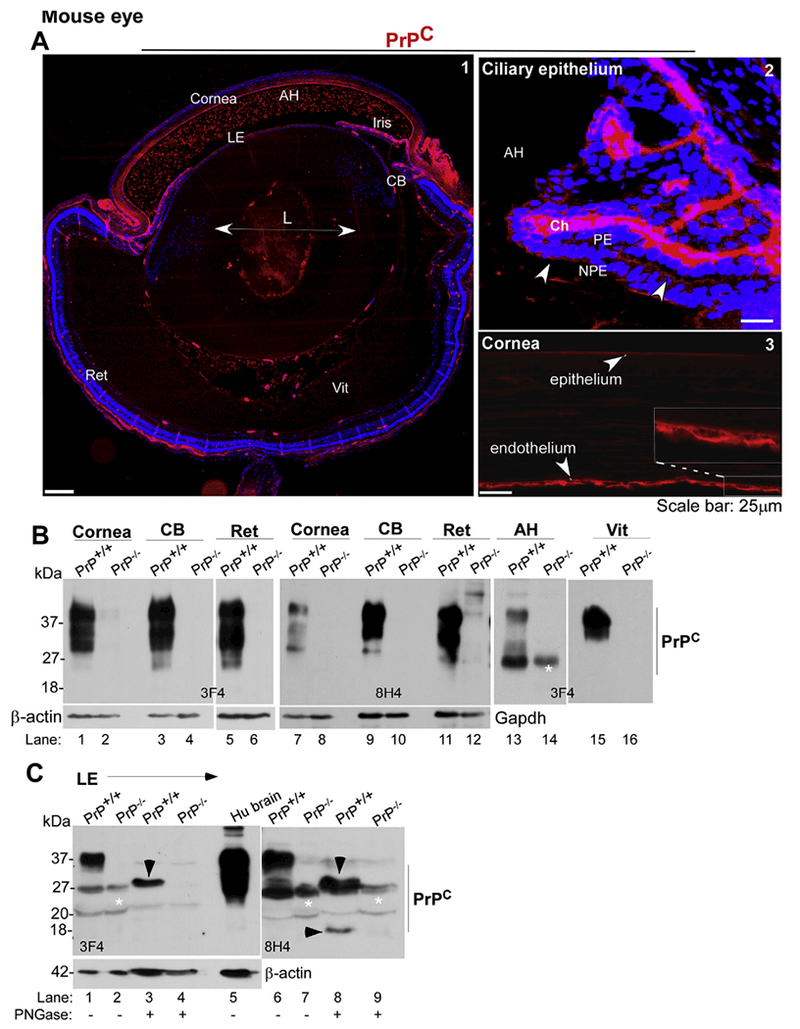
Distribution of PrP^C^ in the mouse eye: **(A)** Immunoreactions of whole eye sections from 9 to 13 day old PrP^+/+^ mouse with 3F4 shows a positive reaction for PrP^C^ in the cornea, iris, CB, lens epithelium, and the retina (panel 1). Punctate reactivity in the anterior chamber is also noted (panel 1). High resolution images of the CB show expression of PrP^C^ on the apical and basolateral surface of NPE cells (panel 2). The cornea shows PrP^C^-specific reaction in the corneal epithelium and endothelium (panel 3). No reaction is noted in PrP^−/−^ samples as expected (negative control in Supplementary data, Fig. 1). Scale bar: 500 μm for panel 1, and 25 μm for panels 2 & 3. **(B)** Probing of pooled lysates from the cornea, CB, and retina, and pooled samples of AH and vitreous from PrP^+/+^ mice with 3F4 and 8H4 shows the expected glycoforms of PrP^C^. No reaction is observed in PrP^−/−^ samples as expected (lanes 1–16). The membranes were reprobed for β-actin (lanes 1–6) and Gapdh (lanes 7–12) as a loading control. **(C)** Probing of pooled lysates of lens epithelial cells from PrP^+/+^ mice with 3F4 and 8H4 shows the expected glycoforms of PrP^C^. (lanes 1 & 6). Deglycosylation shows FL PrP^C^ with 3F4, and an additional α-cleaved Cl form with 8H4 (lane 8). No reactivity is seen with PrP^−/−^ samples (lanes 2, 4, 7, 9). *Non-specific bands. Human brain lysate was fractionated in parallel as a positive control (lane 5). Reaction for β-actin provides a loading control (lanes 1–5). Full images of cropped blots are included in the Supplementary Data.

To determine the expression of PrP^C^ in the mouse eye, fixed sections of PrP^+/+^ mouse eye were immunoreacted with 3F4 and imaged (Fig. 6 A). A positive reaction for PrP^C^ was obvious in the CB, corneal epithelium and endothelium, lens epithelium, and outer retina. Punctate reactivity was also detected in the AH and the vitreous (Fig. 6 A, panel 1). Higher resolution images revealed PrP^C^ reactivity on the basolateral membrane of NPE cells and apical membrane of PE and NPE cells as in human and bovine samples (Fig. 6 A, panel 2). In corneal endothelial cells, PrP^C^ was localized to the plasma membrane (Fig. 6 A, panel 3). No reaction was detected in PrP^−/−^ samples processed in parallel (negative control staining in Fig. 1-Supplementary data).

Evaluation of lysates from PrP^+/+^ and matched PrP^−/−^ controls by Western blotting followed by probing with 3F4 and 8H4 confirmed the expected glycoforms of PrP^C^ in the cornea, CB, retina, AH, and the vitreous of PrP^+/+^ samples, and no reactivity with PrP^−/−^ samples as expected (Fig. 6 B, lanes 1–16). Likewise, probing of lysates from lens epithelial cells with 3F4 and 8H4 revealed the expected glycoforms of PrP^C^ in PrP^+/+^ samples that co-migrated with PrP^C^ from human brain, and a non-specific band in PrP^−/−^ samples. Deglycosylation resulted in the generation of a faster migrating band consistent with full-length PrP^C^ when probed with 3F4, and an additional α-cleaved C-terminal fragment Cl when probed with 8H4. No reactivity was detected in PrP^−/−^ samples as expected (Fig. 6 C, lanes 1–9).

**Fig. 7.**
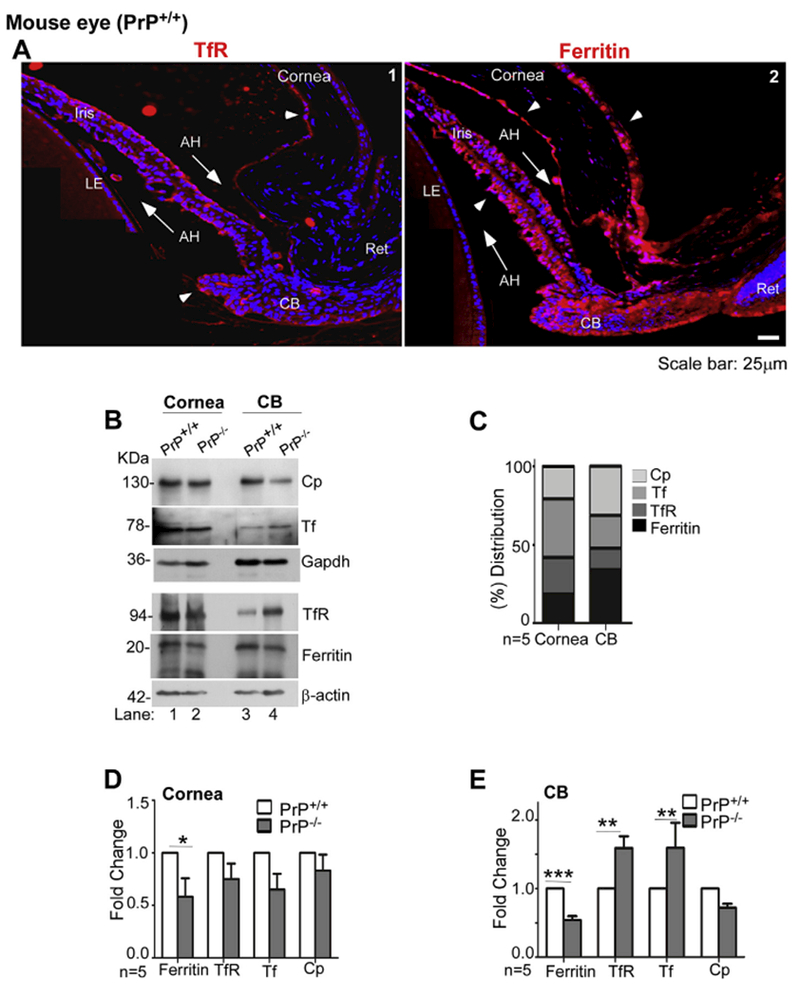
PrP^C^ modulates iron homeostasis in the anterior segment: **(A)** Immunostaining of the anterior segment of PrP^+/+^ mouse eye for TfR shows a positive reaction in the CB, anterior surface of the iris, and corneal endothelium (Cor en) (panel 1). Reactivity for ferritin is more widespread, and is evident in the CB, iris, corneal endothelium, and corneal epithelium (Cor ep) (panel 2). Scale bar: 25 μm. **(B)** Probing of immunoblots from pooled lysates of cornea and CB of PrP^+/+^ and PrP^−/−^ mice for Cp, Tf, TfR, and ferritin shows robust reaction for all proteins in both mouse lines (lanes 1–4). However, absence of PrP results in downregulation of ferritin in the cornea (lanes 1 & 2), and down-regulation of ferritin and Cp and upregulation of TfR in the CB (lanes 3 & 4). Gapdh and β-actin provide a loading control. The β-actin and Gapdh used in Fig. 7B is same as in Fig. 6B as the membranes were reprobed. **(C)** Percentage distribution of each protein in the cornea and CB shows relatively higher expression of Tf and TfR in the cornea, and Cp and ferritin in the CB. **(D)** & **(E)** Quantitation by densitometry following normalization with Gapdh and β-actin shows significant downregulation of ferritin in the cornea, and downregulation of ferrin and Cp and upregu; lation of TfR in the CB of PrP^−/−^ mice relative to PrP^+/+^ controls. Values are mean ± SEM of the indicated n. *p < 0.05, **p < 0.01, ***p < 0.001. Full images of cropped blots are included in the Supplementary Data.

Evaluation of iron transport proteins in the anterior segment of PrP^+/+^ revealed the expression of TfR on NPE cells and corneal endothelial cells (Fig. 7 A, panel 1). Reaction for ferritin was more wide-spread, and was detected in the CB, corneal endothelium and epithelium, and the iris (Fig. 7 A, panel 2).

To evaluate whether PrP^C^ influences iron homeostasis in the AS, protein lysates pooled from the cornea and CB of 15 age-matched PrP^+/+^ and PrP^−/−^ mice were evaluated for the expression of Cp, Tf, TfR, and ferritin (Fig. 7 B). Percentage distribution of these proteins showed ferritin and Cp as major proteins in the CB, while Tf and TfR were predominant in the cornea (Fig. 7 C).

Comparative expression of ferritin TfR, Tf, and Cp revealed significantly reduced expression of ferritin in the cornea of PrP^−/−^ relative to PrP^+/+^ controls, indicating a phenotype of relative iron deficiency (Fig. 7 D). Likewise, the CB from PrP^−/−^ mice showed significant downregulation of ferritin and Cp and upregulation of TfR relative to PrP^+/+^ controls, indicating iron deficiency (Fig. 7 E). Similar observations were noted in the cornea, CB and retina of PrP^−/−^ mice relative to PrP^+/+^ controls expressing wild-type levels of PrP^C^ (data not shown), ruling out the possibility that these observations are an artifact of transgene expression.

Together, the above results demonstrate active involvement of PrP^C^ in iron transport from the CB to the AH, and uptake by the cornea.

### 3.4. Mouse models of chronic wasting disease show aggregation of PrP^C^ in the cornea and CB

**Fig. 8.**
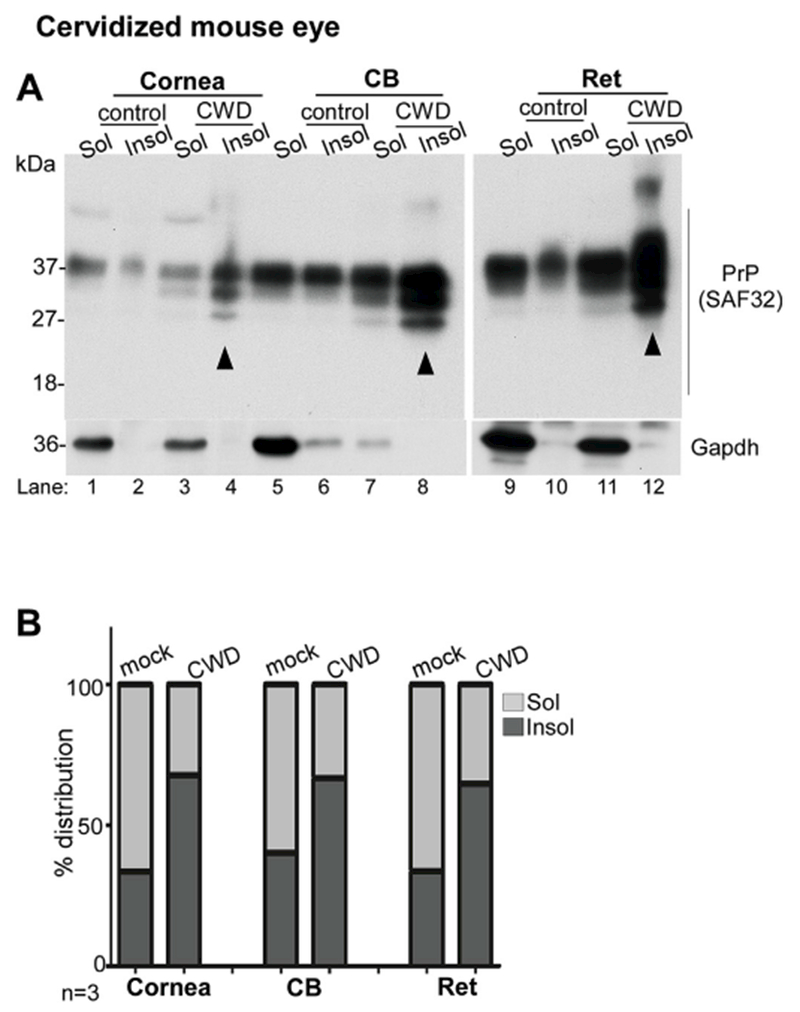
CWD infected mice show aggregation of PrP^C^ in the retina, cornea and CB: **(A)** Detergent soluble (sol) and insoluble (insol) fractions from pooled lysates of the cornea (cor), CB, and retina (ret) of mock and CWD infected mice were analyzed by Western blotting. Probing with SAF32 shows partitioning of majority of PrP^C^ in the retina, cornea, and CB of mock-infected animals in the soluble fraction (lanes 1,5,9). In CWD-infected samples, on the other hand, majority of PrP^C^ in the retina, cornea, and the CB partitions in the detergent-insoluble phase (lanes 4, 8, 12). Gapdh provides a loading control, and partitions in the detergent-soluble phase as expected. **(B)** Percent distribution of PrP^C^ in the soluble vs. insoluble fractions shows relatively more insoluble PrP^C^ in the CWD-infected cornea, CB, and retina relative to mock controls. Full images of cropped blots are included in the Supplementary Data.

To evaluate whether the PrP^C^ expressed in the cornea is susceptible to prion infection, transgenic mice expressing cervid PrP^C^ (Kong et al., 2005) were inoculated with CWD-infected brain homogenate intracerebrally, and PrP^C^ in the cornea and CB was evaluated for a possible change in conformation to the aggregated, PrP-scrapie isoform. Accordingly, pooled lysates from the cornea, CB, and retina of 10 CWD-infected clinically sick mice and an equal number of mock controls were subjected to differential centrifugation to isolate detergent-soluble and insoluble fractions. Both fractions were fractionated by SDS-PAGE and transferred proteins were probed with SAF32. Consistent with the involvement of the retina in all prion disorders (Armstrong, 2006), a significant amount of PrP^C^ in the retina of CWD-infected animals partitioned in the detergent-insoluble phase, while most of the PrP^C^ in mock-infected samples was detected in the soluble phase (Fig. 8, lanes 9–12). Surprisingly, the cornea and CB of CWD-infected animals also showed significant detergent-insoluble PrP^C^, suggesting a conformational change to the aggregated, disease-causing PrP-scrapie isoform (Fig. 8, lanes 1–8). Quantification by densitometry confirmed a significant increase in insoluble PrP^C^ in CWD-infected samples relative to mock controls (Fig. 8 B). The conventional proteinase-K resistance of detergent-insoluble fraction to confirm the presence of PrP-scrapie could not be performed due to the small sample size from mice. Nevertheless, these observations suggest that PrP^C^ in the cornea and CB of CWD infected animals is likely to carry scrapie infectivity.

## 4. Discussion

This report provides evidence that the ciliary epithelium regulates iron homeostasis in the anterior segment, and PrP^C^ contributes to this process. The relative expression of major iron modulating proteins suggests efflux by the ciliary epithelium, and uptake by the corneal endothelium. Widespread expression of PrP^C^ in the anterior segment, predominance of β-cleaved PrP^C^ in the ciliary epithelium, and a phenotype of relative iron deficiency in PrP^−/−^ mice supports active participation of PrP^C^ in iron transport as reported for outer retina and other cell types (Asthana et al., 2017; Haldar et al., 2015; Tripathi et al., 2015). Soluble PrP^C^ in the AH and vitreous has significant clinical implications because of the known interaction between PrP^C^ and amyloid-β (Hirsch et al., 2014; Kang et al., 2013; Lauren, 2014), and requires further exploration.

A tight control of iron homeostasis in the anterior segment is essential to protect the cornea and lens from photo-oxidative damage. Not surprisingly, the concentration of Tf in the AH of diurnal species is much higher than blood plasma, and significantly more than nocturnal species. Moreover, iron saturation of Tf in the AH is 10% as opposed to 30% in the plasma, providing additional buffering capacity against iron (VázQuEz-QuIñoNES and García-Castiñeiras, 2009). Most of the Tf in the AH is secreted by the ciliary epithelium and lens epithelial cells (Delamere, 2005; Garcia-Castineiras, 2010). The source of iron is less clear. Accumulation of iron in the ciliary epithelium of mouse models lacking Cp and hephaestin (Hadziahmetovic et al., 2008, 2011), a hormone that regulates Fpn, suggests active export of iron from these cells (Hadziahmetovic et al., 2008; Ganz, 2013). Strong reactivity for TfR near the apical domain and Fpn and Cp on the basolateral membrane of non-pigmented epithelial cells suggests active export of Tf-iron to the AH. Gene expression studies documenting the expression of Fpn and hephaestin in human pigmented and non-pigmented epithelial cells further support this conclusion (Janssen et al., 2012, 2013). Higher expression of Cp in the ciliary epithelium relative to TfR suggests a predominant role in iron export rather than import. Abundant presence of Cp in the AH suggests secretion from the ciliary epithelium and lens epithelium (Harned et al., 2006), facilitating oxidation of Fe^2+^ exported through Fpn to Fe^3+^ for conjugation with Tf.

Corneal endothelial cells, on the other hand, expressed higher levels of TfR relative to Cp, suggesting import of iron as the major activity. Since the cornea is avascular, all nutrients, including iron, are acquired from the AH across these cells. Although the polarity of protein expression and direction of iron transport is difficult to judge from our data, it is plausible that TfR and Fpn are functionally versatile, and are transported to the apical or basolateral domain in response to the labile iron pool of endothelial cells. The relatively higher expression of Fpn relative to TfR on Corneal epithelial cells, on the other hand, suggests active export of iron to the tear film. Absence of Fpn reactivity on bovine corneal epithelium was unexpected, and requires further exploration.

The expression of PrP^C^ on pigmented and non-pigmented epithelial cells was not entirely unexpected given their origin from the neural crest (Centanin and Wittbrodt, 2014; Delamere, 2005). As in retinal pigment epithelial cells, PrP^C^ was localized to the basolateral membrane of pigmented cells facing the choroid plexus (Asthana et al., 2017). In non-pigmented epithelial cells, a continuation of the neural retina (Delamere, 2005), PrP^C^ was detected in intracellular vesicles and the basolateral membrane facing the AH. A more robust immunoreaction with the N-terminal antibody (SAF32) relative to 3F4 suggested the presence of mainly full-length PrP^C^ in non-pigmented cells in contrast to the β-cleaved form in pigmented cells. A similar orientation of PrP^C^ has been reported in retinal pigment epithelial cells where β-cleavage is associated with transport of iron through DMT1 and ZIP14 (Asthana et al., 2017). Interestingly, gene profiling studies have identified ZIP14 and ZIP8 in human pigmented and non-pigmented epithelial cells (Janssen et al., 2013), suggesting a similar role of PrP^C^ at this site. Although the precise mechanism by which PrP^C^ facilitates transport of iron to the AH is not clear from our data, a phenotype of relative iron deficiency in the cornea, CB, and retina of PrP^−/−^ mice relative to PrP^+/+^ controls suggests that PrP^C^ plays an essential role as in the outer retina (Asthana et al., 2017).

The expression of PrP^C^ on corneal endothelial and epithelial cells and lens epithelial cells suggests a role specific to polarized monolayers. Since the corneal endothelial cells of PrP^−/−^ mice express significantly less ferritin than PrP^+/+^ controls, it is likely that PrP^C^ facilitates uptake of iron by the Tf/TfR pathway as in other cell types (Baksi et al., 2016). This is an important unanswered question with significant clinical implications since a breach of these monolayers is likely to result in corneal and lens opacities with eventual blindness. Soluble PrP^C^ in the aqueous and vitreous humor is probably released from lining cells, and is likely to play a protective role by binding amyloid-β, a proteolytic product of amyloid precursor protein implicated in the pathogenesis of glaucoma and age related macular degeneration (Anderson et al., 2004; Guillot-Sestier et al., 2009; Prakasam et al., 2010; Ratnayaka et al., 2015; Salazar et al., 2017; Shah et al., 2017). Further investigations are necessary to understand the functional significance of soluble PrP^C^, and strategies to use this information in delaying or preventing ocular disorders associated with increased levels of amyloid-β.

The expression of PrP^C^ in the corneal epithelium raises the possibility of prion transmission through the cornea. However, minimal amounts of PrP-scrapie were detected in the cornea of patients with sporadic or variant Creutzfeldt-Jakob disease, human prion disorders, leading to the classification of cornea as low risk in comparison to the retina and optic nerve that were considered highly infectious (Armitage et al., 2009; Armstrong, 2006; Head et al., 2003). However, transmission of prion disease by conjunctival instillation of PrP-scrapie has been demonstrated in experimental mice and sheep (Gonzalez et al., 2014; Scott et al., 1993). Although the proposed route to the brain is through blood, perhaps via lacrimal glands (Gonzalez et al., 2014), our observations indicate contiguous spread from the cornea to the retina and optic nerve as a distinct possibility. Efficient transport of CWD prions in the opposite direction, i.e. from the brain to the cornea, support the above conclusion. Such a possibility has serious implications for the horizontal transmission of CWD in cervids through corneal abrasions, a likely possibility while grazing.

## 5. Conclusions

In conclusion, this study demonstrates independent regulation of iron homeostasis in the anterior segment by the ciliary epithelium, and a prominent role of PrP^C^ in this process. Identification of soluble PrP^C^ in the aqueous and vitreous humor provides a unique opportunity to sequester amyloid-β in these compartments, a challenging but therapeutically rewarding task for chronic disorders such as glaucoma and age-related macular degeneration. And finally, horizontal transmission of CWD through corneal abrasions is a distinct possibility and requires further exploration.

## Supplementary Material

Supplemental file

## Nomenclature

AH: aqueous humor
CB: ciliary body
PE: pigmented epithelium
NPE: non-pigmented epithelium
PrP^C^: prion protein
Prp^−/−^: PrP-knockout
Tf: Transferrin
TfR: transferrin receptor
Fpn: ferroportin
Cp: ceruloplasmin
CWD: chronic wasting disease
