# Supplementary material for: Prion protein modulates iron transport in the anterior segment: Implications for ocular iron homeostasis and prion transmission
Source: Exp Eye Res. Author manuscript; Available in PMC 2018 Oct 1. (PMC6167182; doi:10.1016/j.exer.2018.05.031)
Supplement: Supplemental file [file NIHMS982379-supplement-Supplemental_file.doc]

**Supplementary Data**

**Control staining for Figures 1(A-B); Figures 2(A-B); Figures 4(C-D); Figure 5A; and Figure 7A**


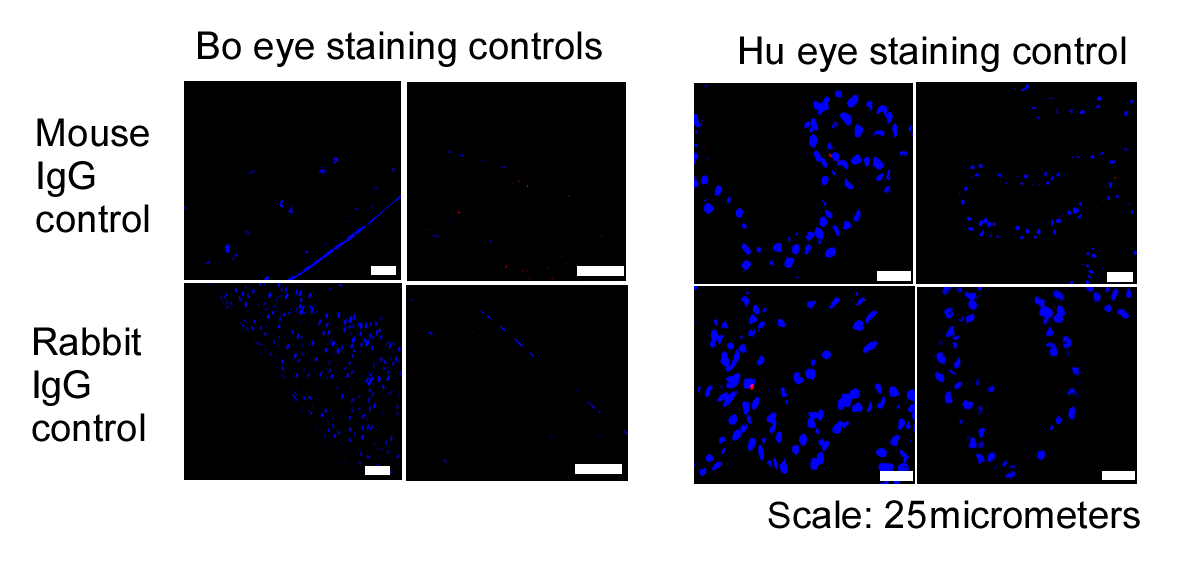
 **Negative control of PrP staining of mouse eye for Figure 6A and H&E stain of mouse eye.**

**
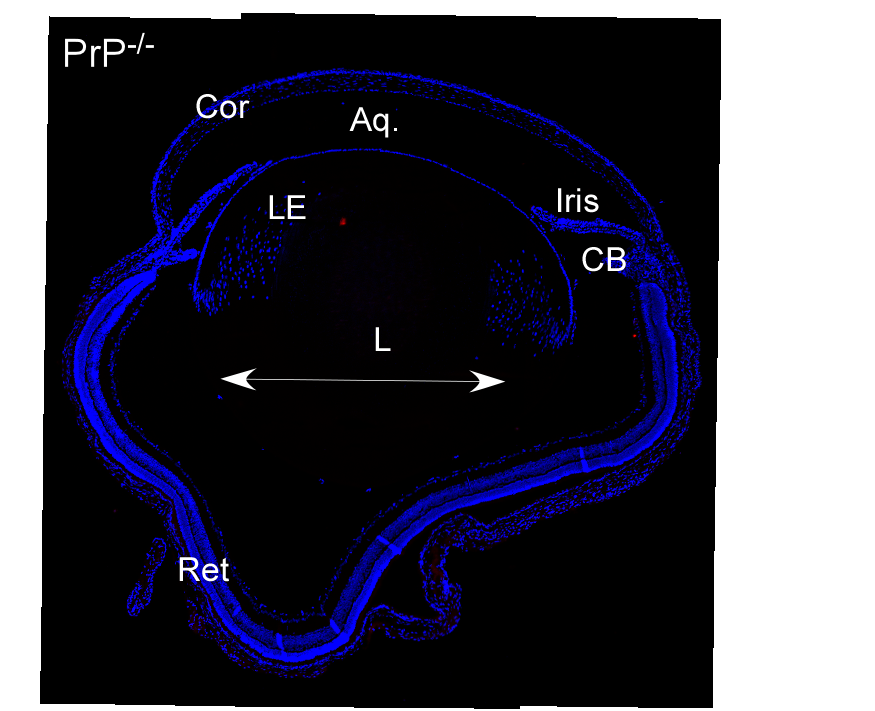

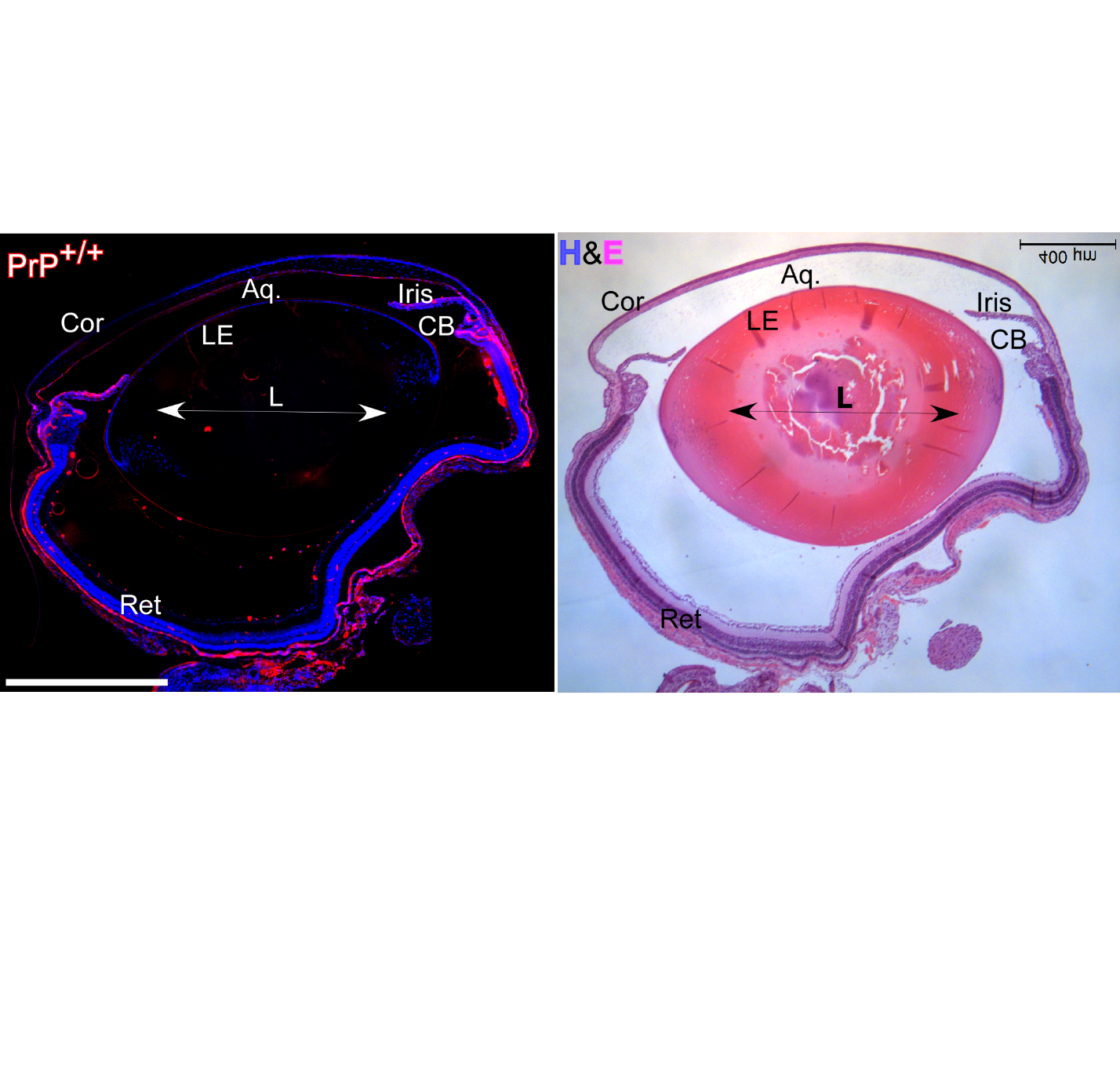
**

Scale: 100 micrometers

Mouse

Eye

Scale: 400 micrometers

**Below, we show complete gels of cropped protein bands. Areas included in the Figures are marked with black boxes. Samples not relevant to this study are not labeled.**

**Figure 3**

**Figure 3A-The membranes were reprobed:** Order of probing:1) 8H4 2) Ferritin, 3) TfR, 4) Tf, 5) Gapdh (represented in Figure3B), 6) Cp

**Cp**

**
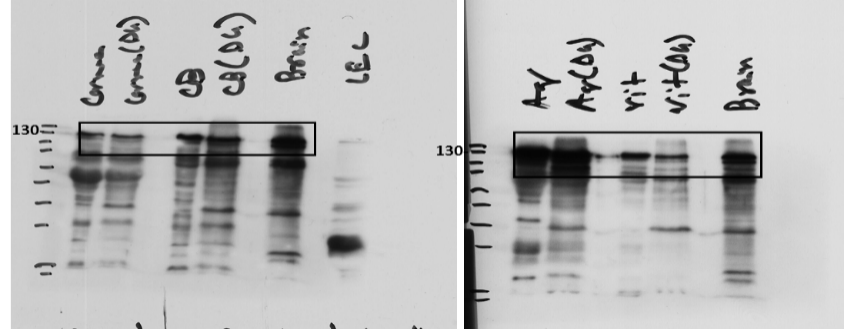

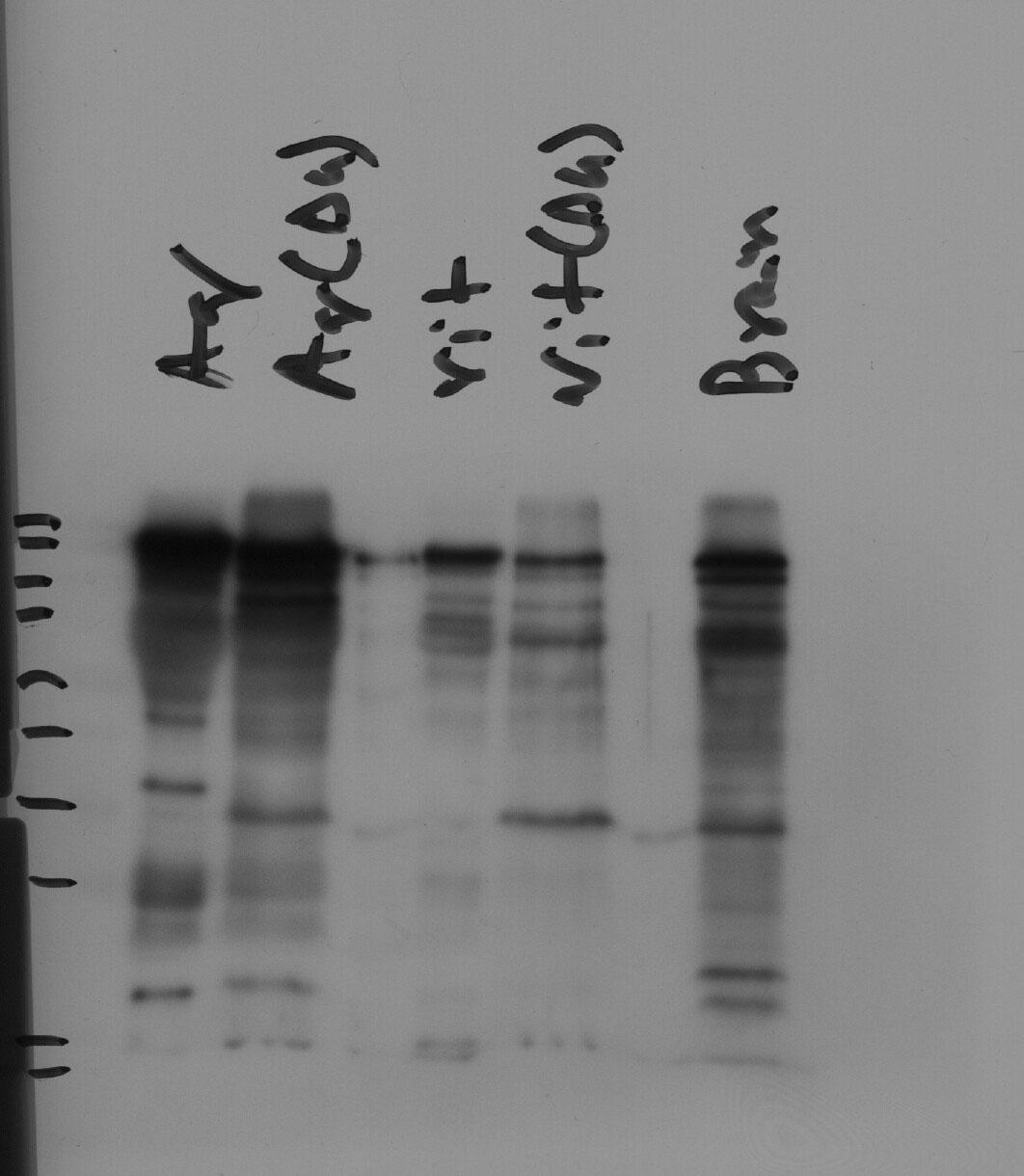
**

130

130

Lane:1 2 3 4 5

Lane: 6 7 8 9

**TfR**

b.cor b.cb hu brain

b.Aq hu b.vit hu

**
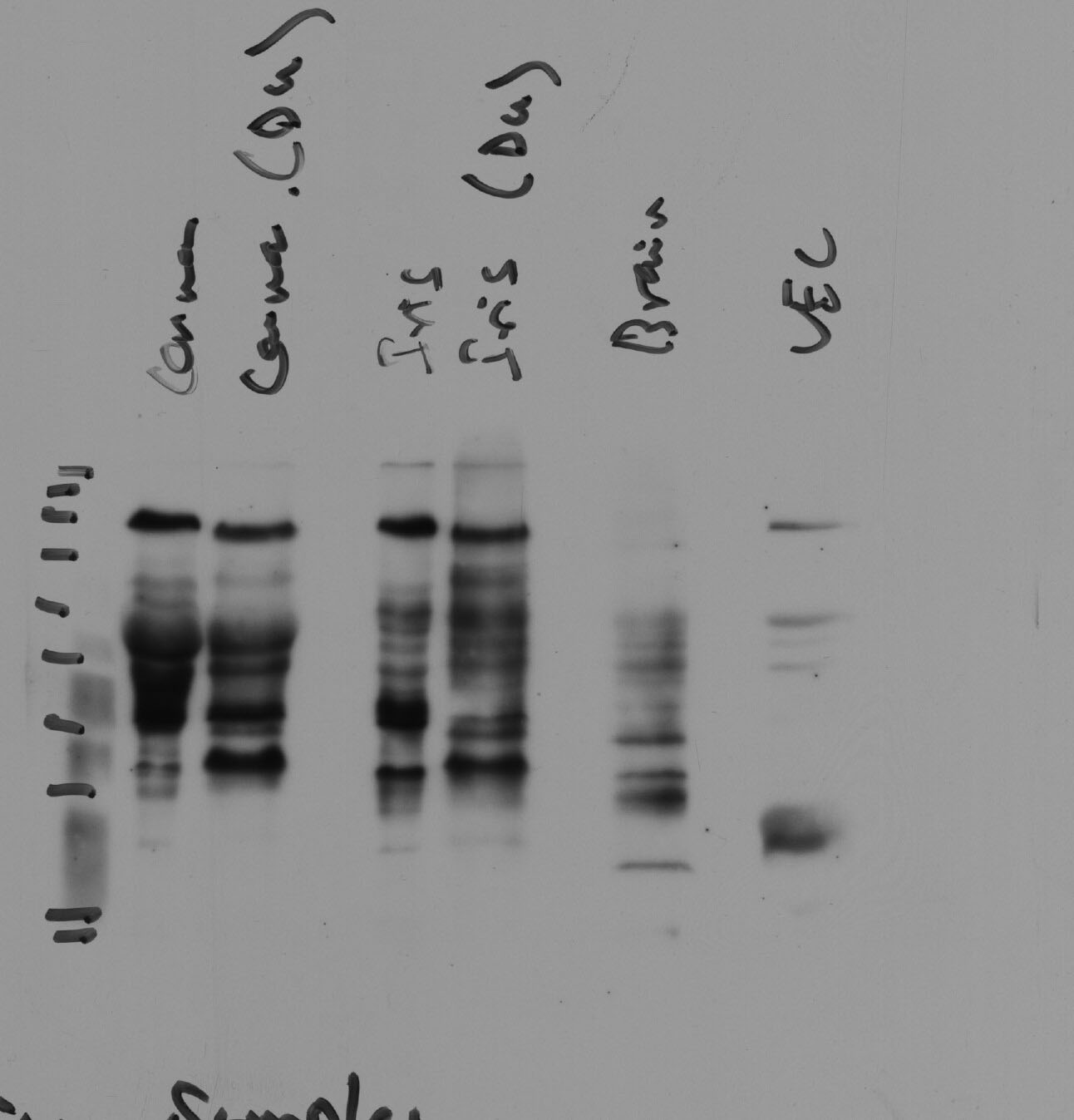

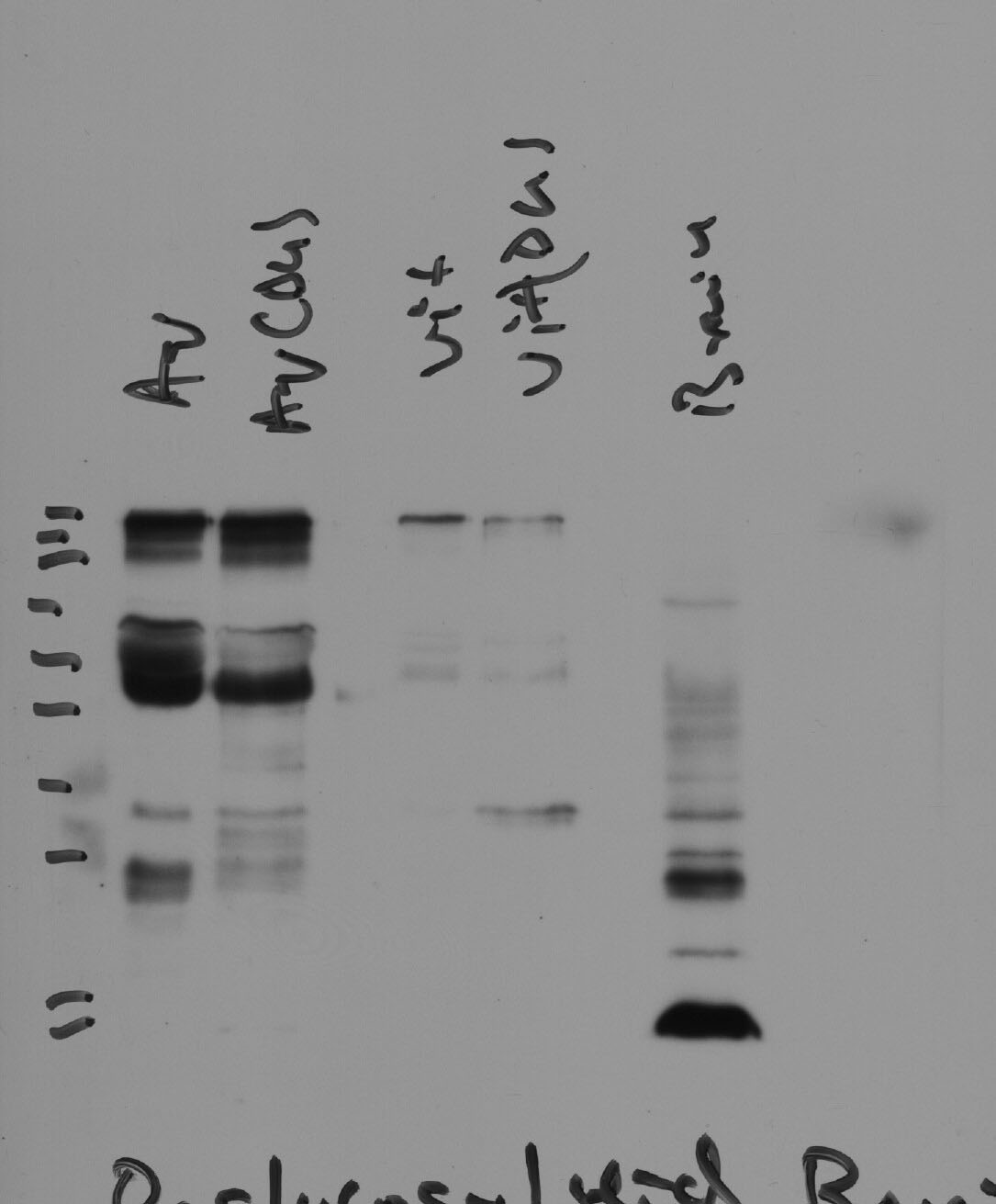
**

94

94

Lane:1 2 3 4 5

Lane:6 7 8 9

**Tf**

b.cor b.cb hu brain

b.Aq hu b.vit hu

**
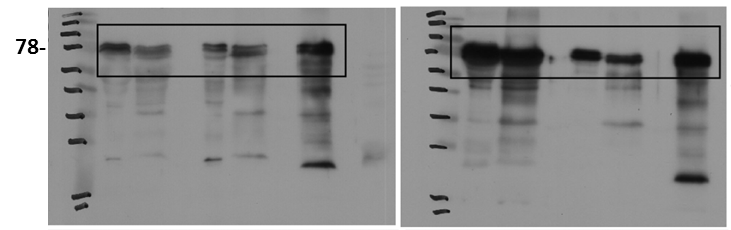
**  **
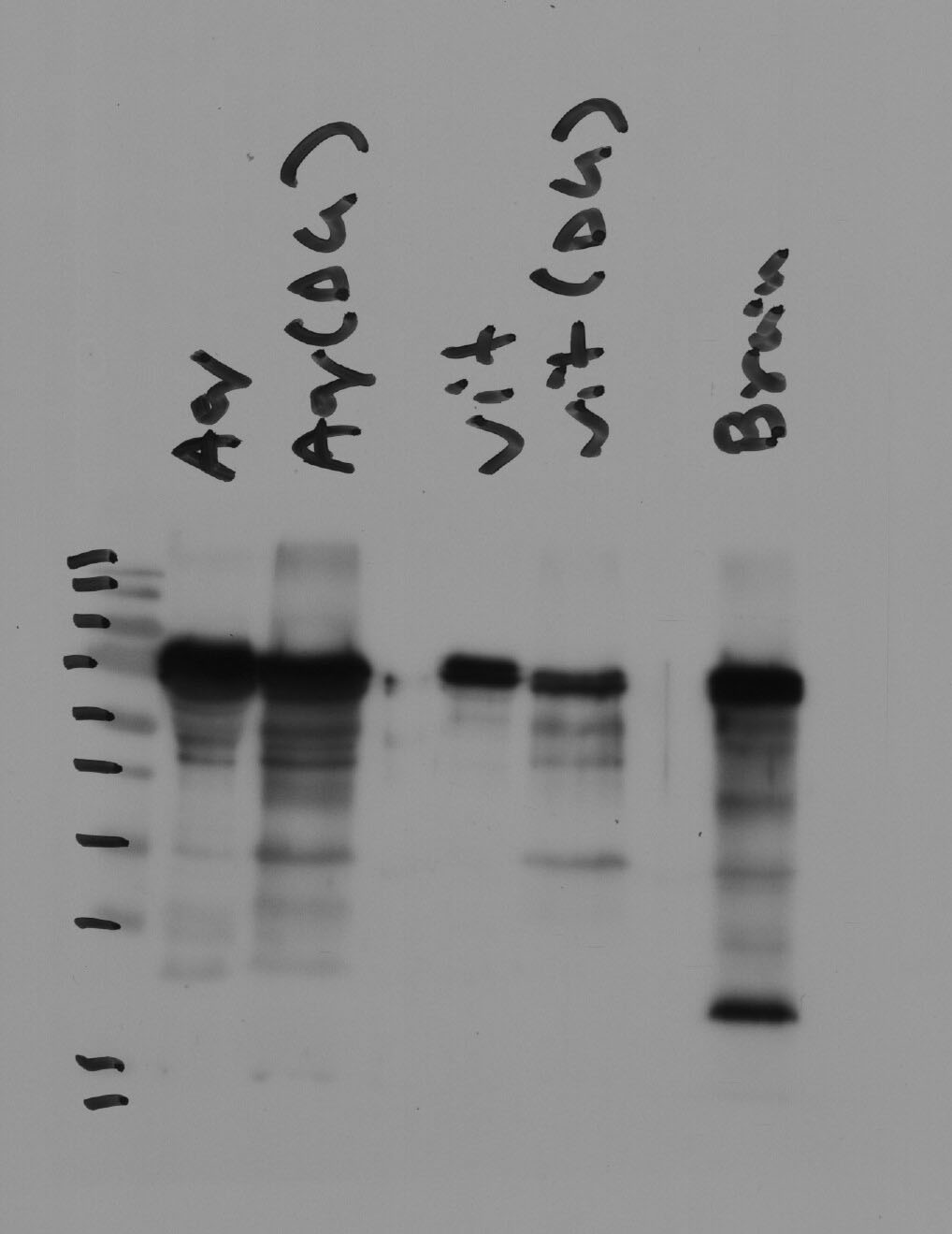
**

78

78

Lane: 1 2 3 4 5

Lane: 6 7 8 9

**Ft**

b.cor b.cb hu brain

b.Aq hu b.vit hu

**
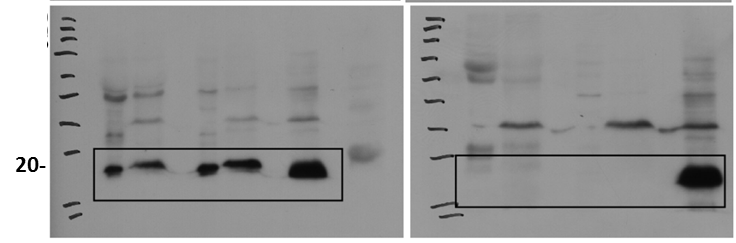
**  **
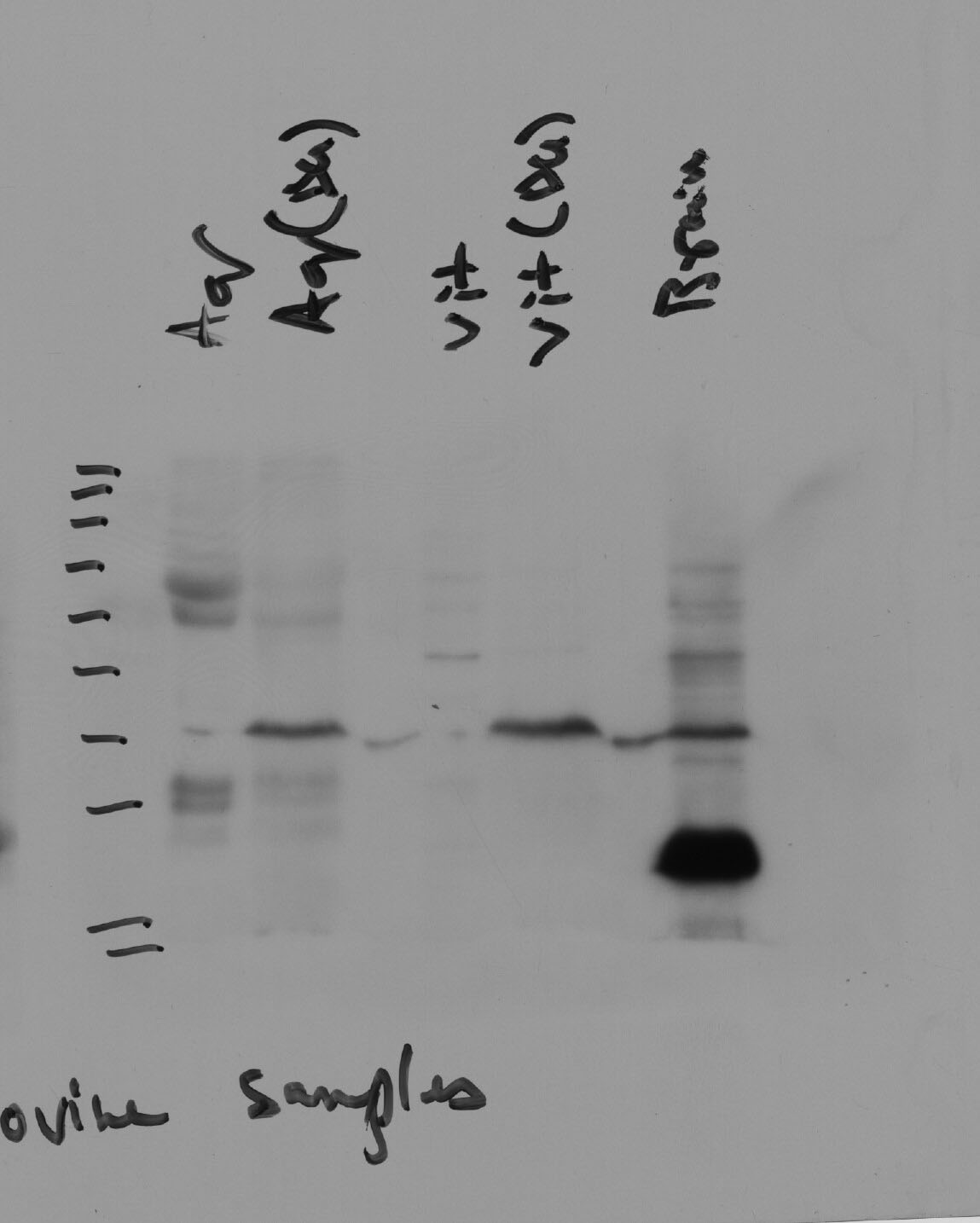
**

20

20

Lane: 1 2 3 4 5

Lane: 6 7 8 9

**Gapdh**

b.cor b.cb hu brain

**
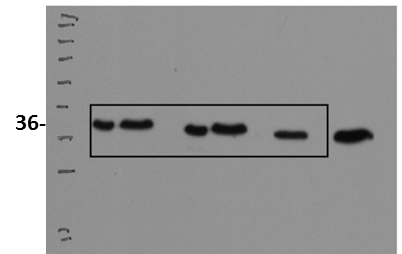
**

Lane: 1 2 3 4 5

**Figure 3D-The membranes were reprobed**

**Fpn**

**
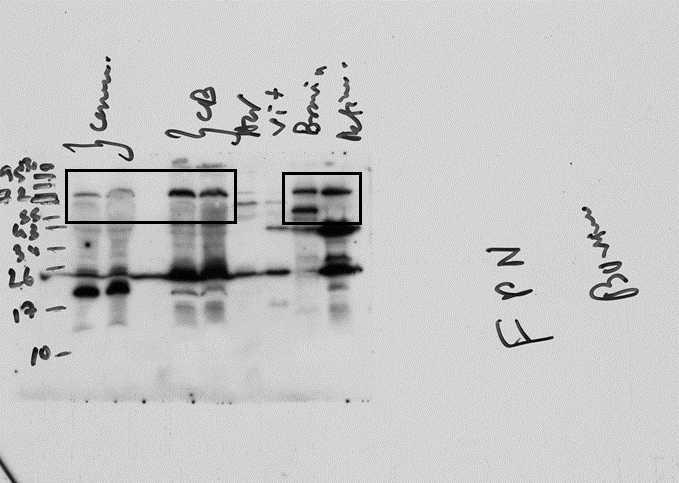
Figure 3D-**Order of probing: 1) Fpn

Lane: 1 2 3 4 5 6

**Gapdh**

**
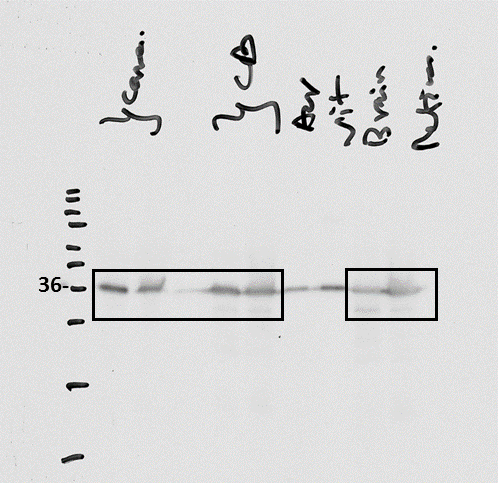
Figure 3D-**Order of probing: 1) Fpn, 2) Gapdh

Lane: 1 2 3 4 5 6

**Figure 5**

**Figure 5B-The membranes were reprobed**

**8H4**

**b. cor**

**20µg 40µg**

**
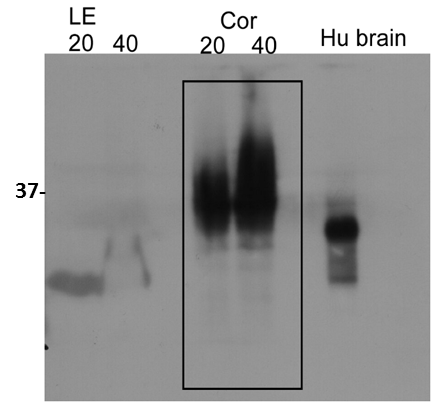
Figure 5B-**Order of probing: 1)8H4

Lane: 1 2

**β actin**

**20µg 40µg**


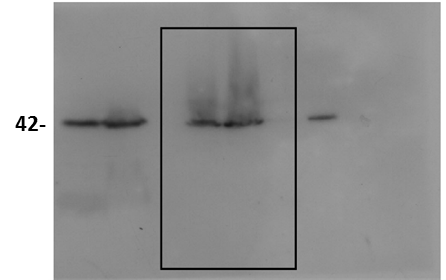
**Figure 5B-**Order of probing: 1) 8H4, 2) β actin

Lane: 1 2

**b.CB**

**20µg40µg hu brain**


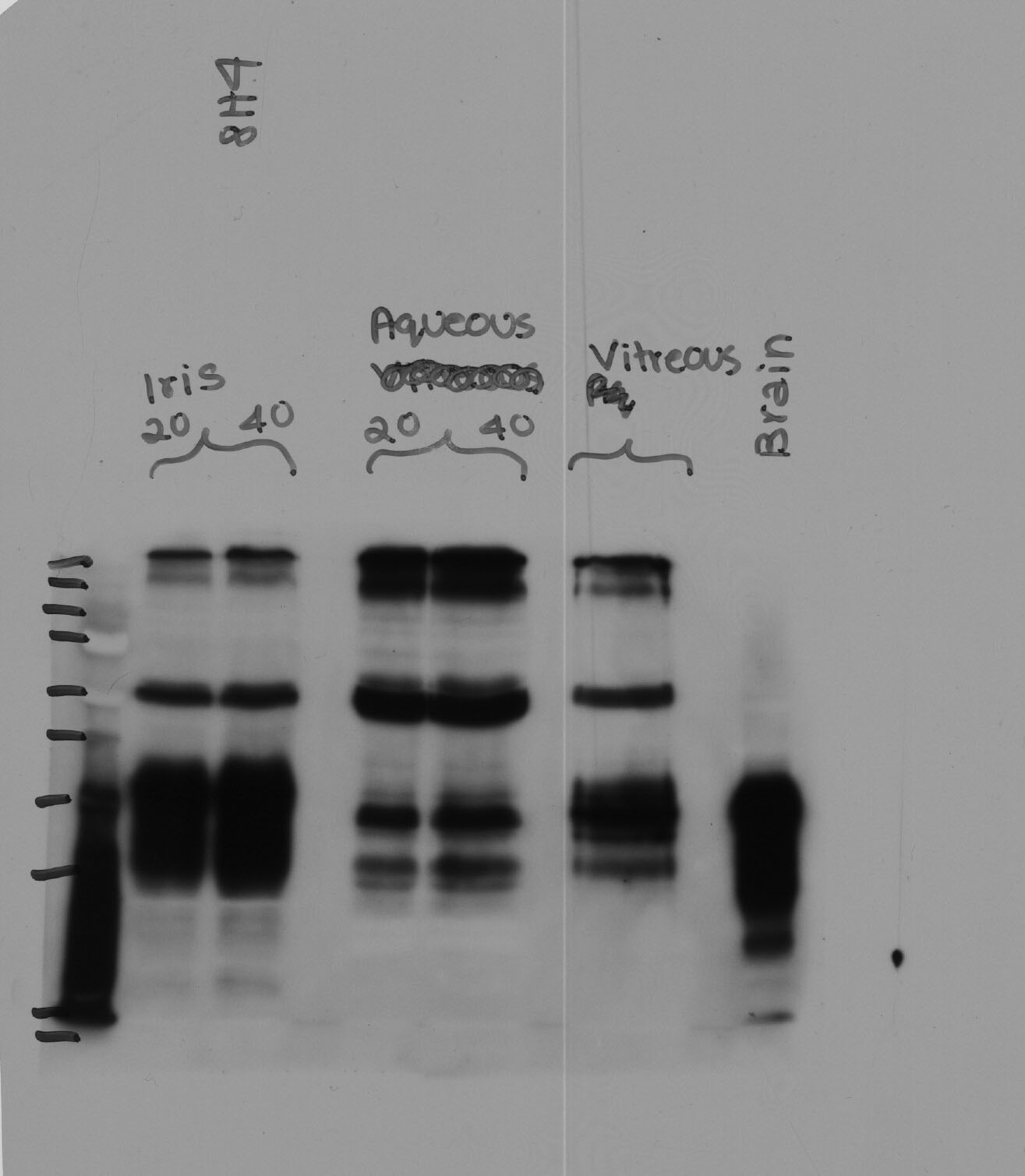
 **Figure 5B-**Order of probing: 1) 8H4

37

Lane: 3 4

**β actin**

**20µg40µg hu brain
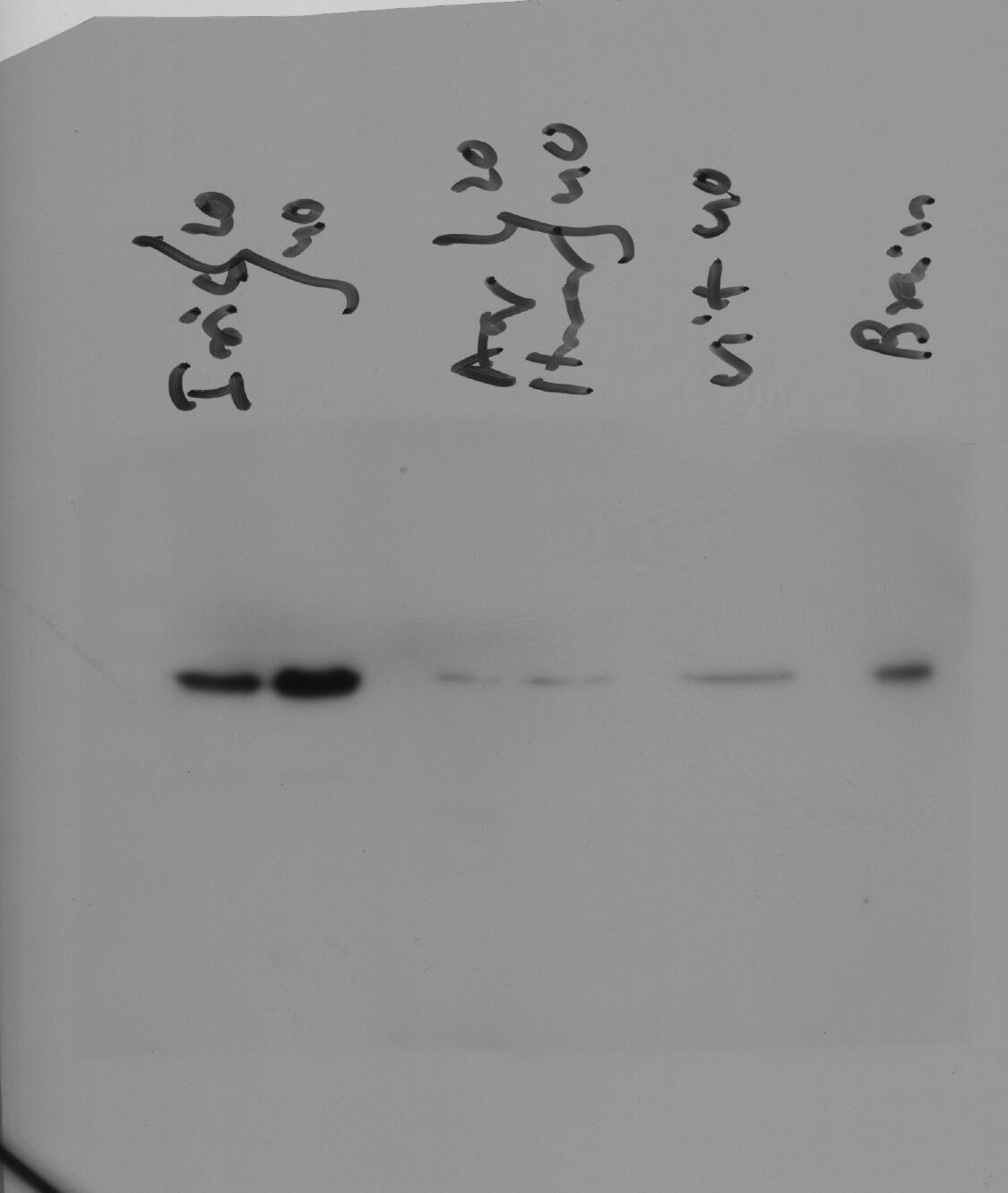
** **Figure 5B-**Order of probing: 1)8H4, 2) β actin

42

Lane: 3 4

**8H4**


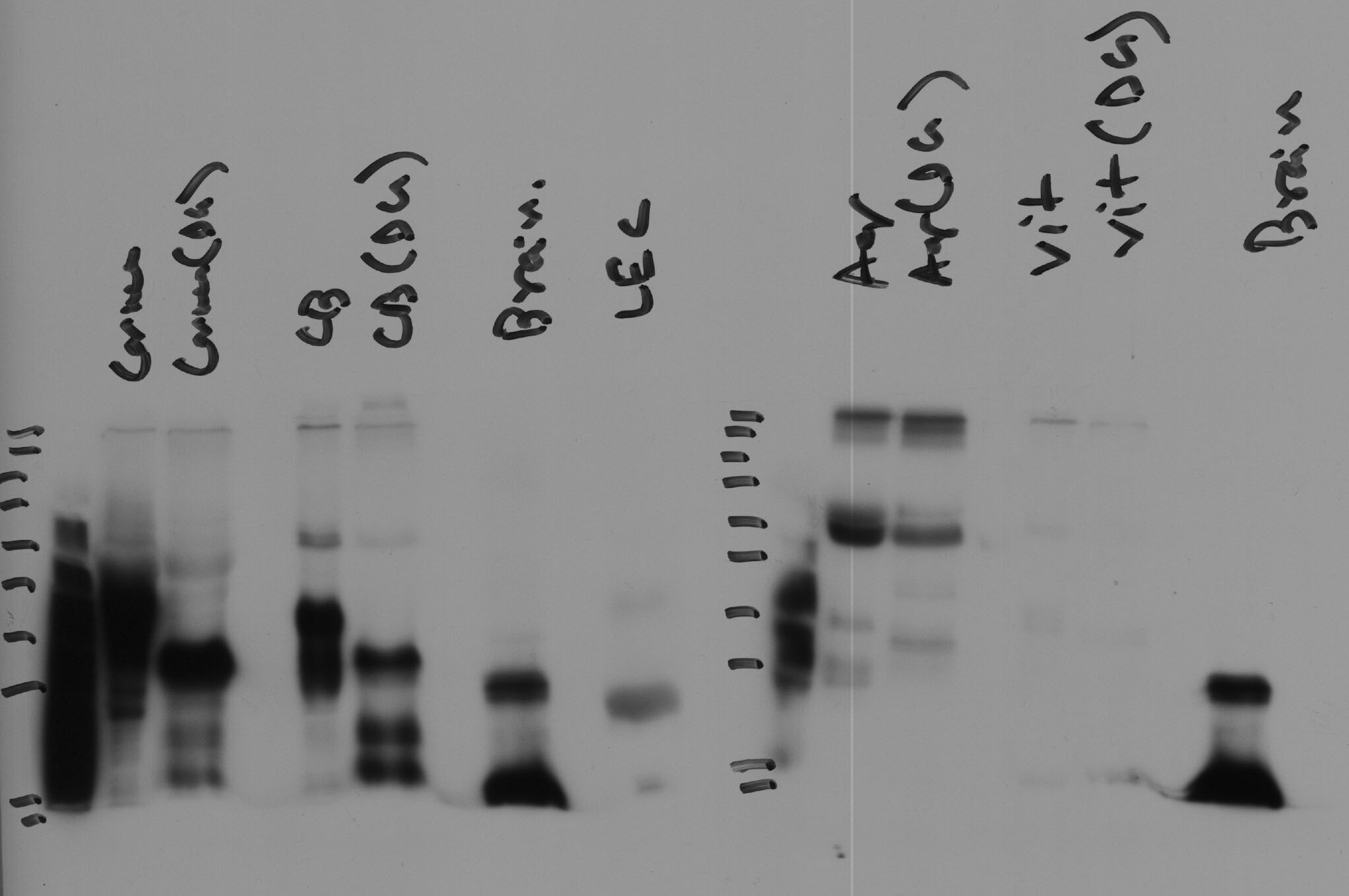
 **Figure 5B-**Order of probing: 1)8H4

Lane: 5 6 7 8

**Gapdh**


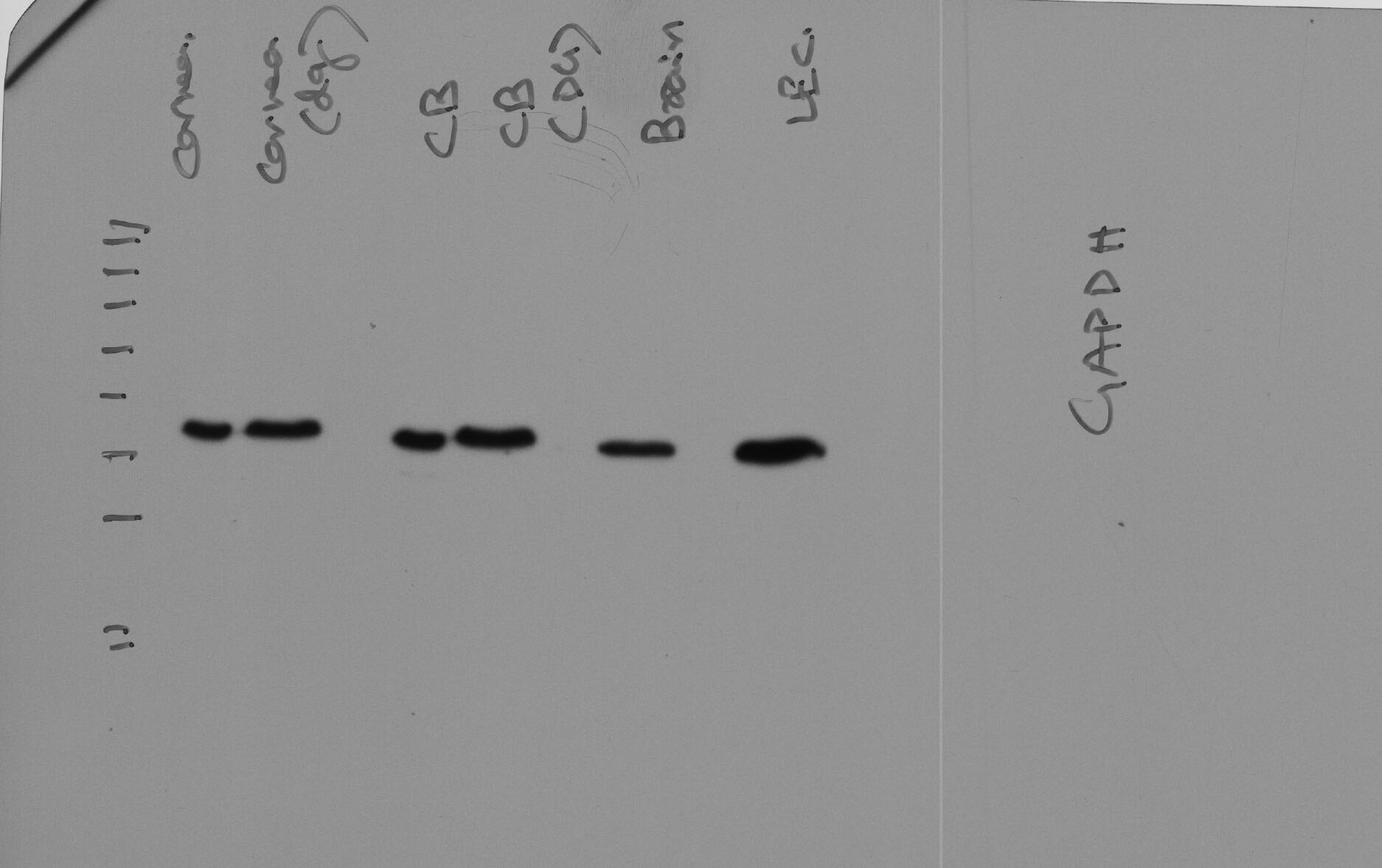
 **Figure 5B-**Order of probing: 1) 8H4, 2) Gapdh,

Lane: 5 6 7 8

**Figure 5D-The membranes were reprobed**

**b.AqH b.VitH**

**b.AqH b.vitH**

**
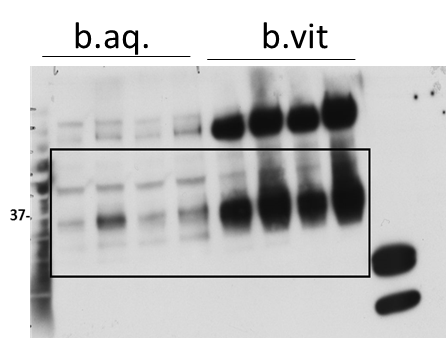
 Figure 5D-**Order of probing: 1) 8H4

Lane: 1 2 3 4 5 6 7 8

**
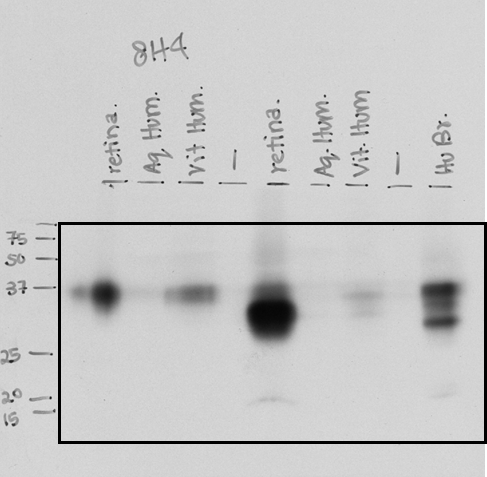
Figure 5D-**Order of probing: 1)8H4

Lane: 9 10 11 12 13 14 15

**
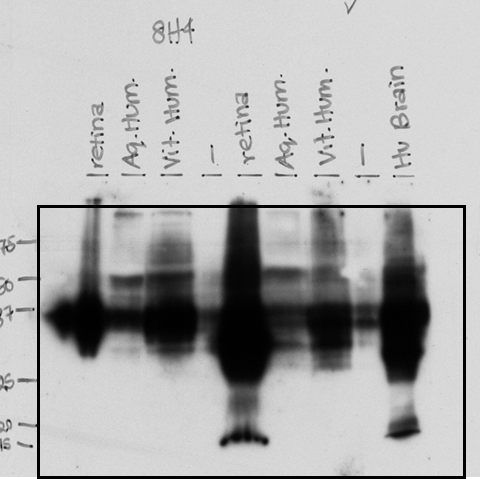
Figure 5D-**Order of probing: 1)8H4

Lane: 9 10 11 12 13 14 15

**Figure 6**

**Figure 6B-The membranes were reprobed**

**3F4**

**
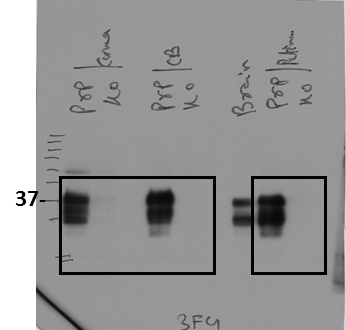
Figure 6B-**Order of probing: 1)3F4

Lane: 1 2 3 4 5 6

**β actin**

**
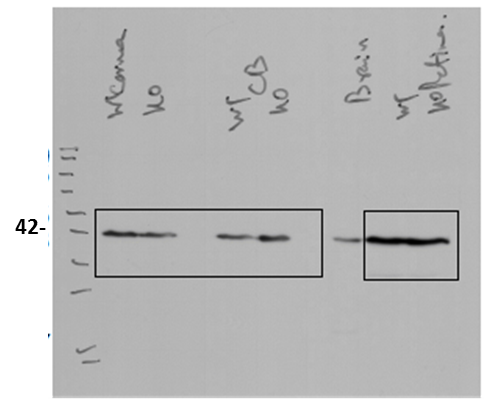
Figure 6B-**Order of probing: 1) 3F4, 2) β actin

Lane: 1 2 3 4 5 6

**8H4**

**
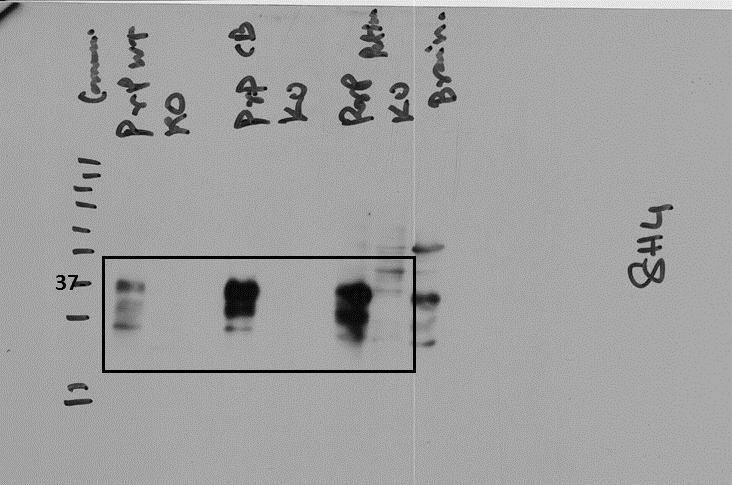
 Figure 6B-**Order of probing: 1)8H4

Lane: 7 8 9 10 1112

**Gapdh**

**
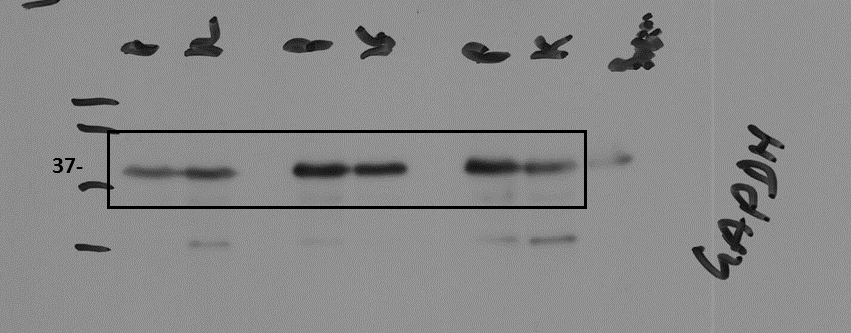
Figure 6B-**Order of probing: 1) 8H4, 2) Gapdh

Lane: 7 8 9 10 1112

**3F4**

**
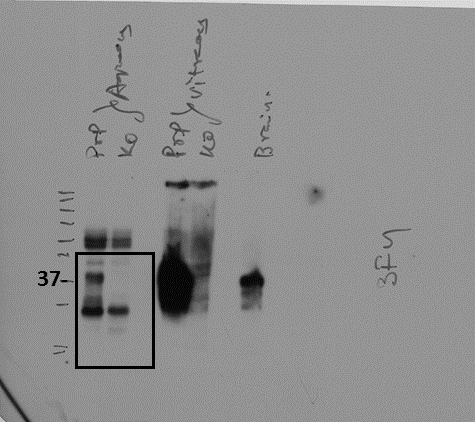
Figure 6B-**Order of probing: 1) 3F4

Lane: 13 14

**3F4**

**
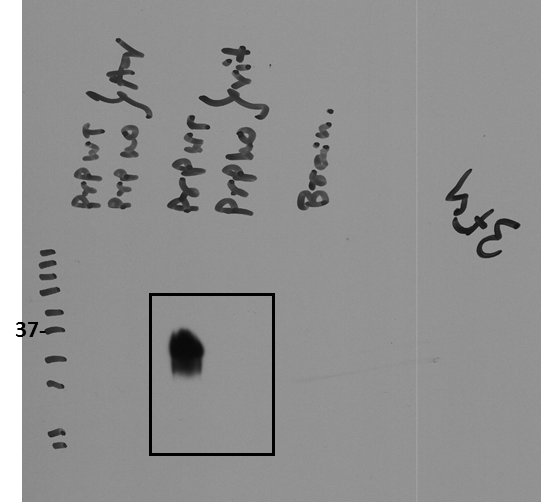
Figure 6B-**Order of probing: 1) 3F4

Lane: 15 16

**Figure 6C-The membranes were reprobed**

**3F4**

**
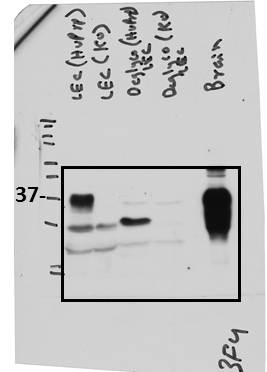
Figure 6C-**Order of probing: 1) 3F4

Lane:1 2 3 4 5

**β actin**

**
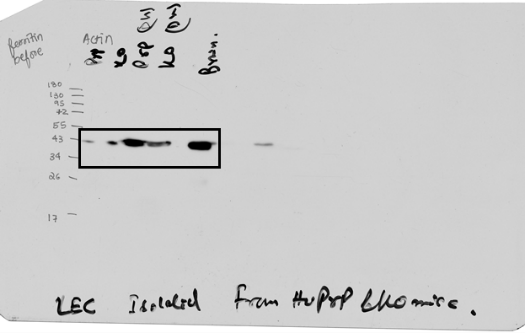
Figure 6C-**Order of probing: 1) 3F4, 2) β actin

42

Lane:1 2 3 4 5

**8H4**


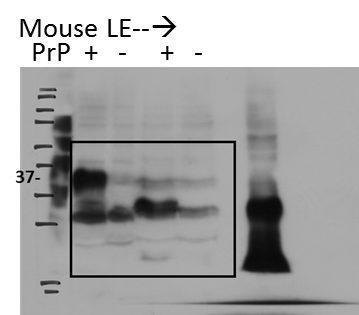
**Figure 6C-**Order of probing: 1) 8H4

Lane: 6 7 8 9

**Figure 7**

**Figure 7B-The membranes were reprobed**

**Cp**


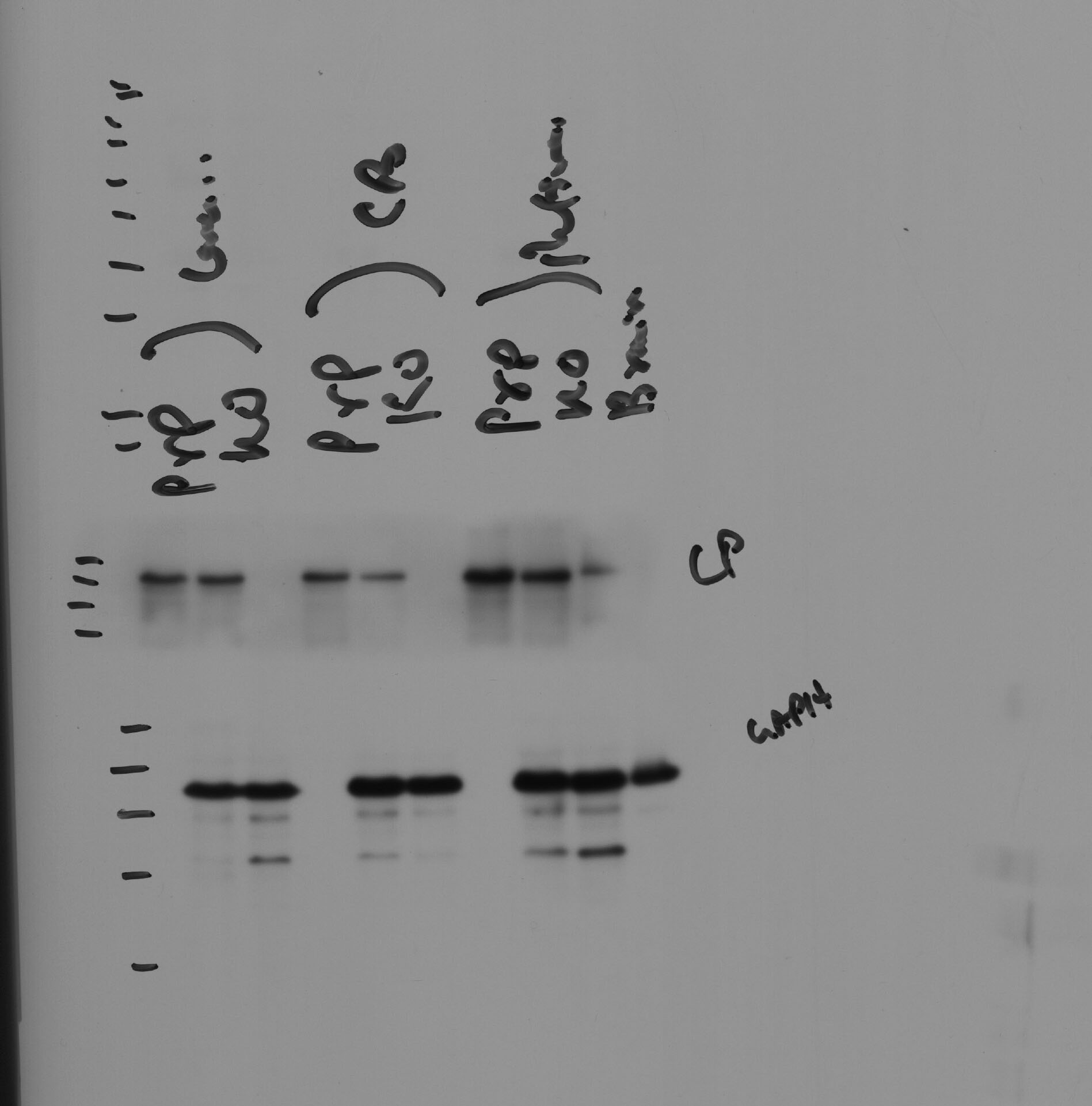
**Figure 7B-**Order of probing: 1) Tf, 2) Cp

130

Lane: 1 2 3 4

**Tf**


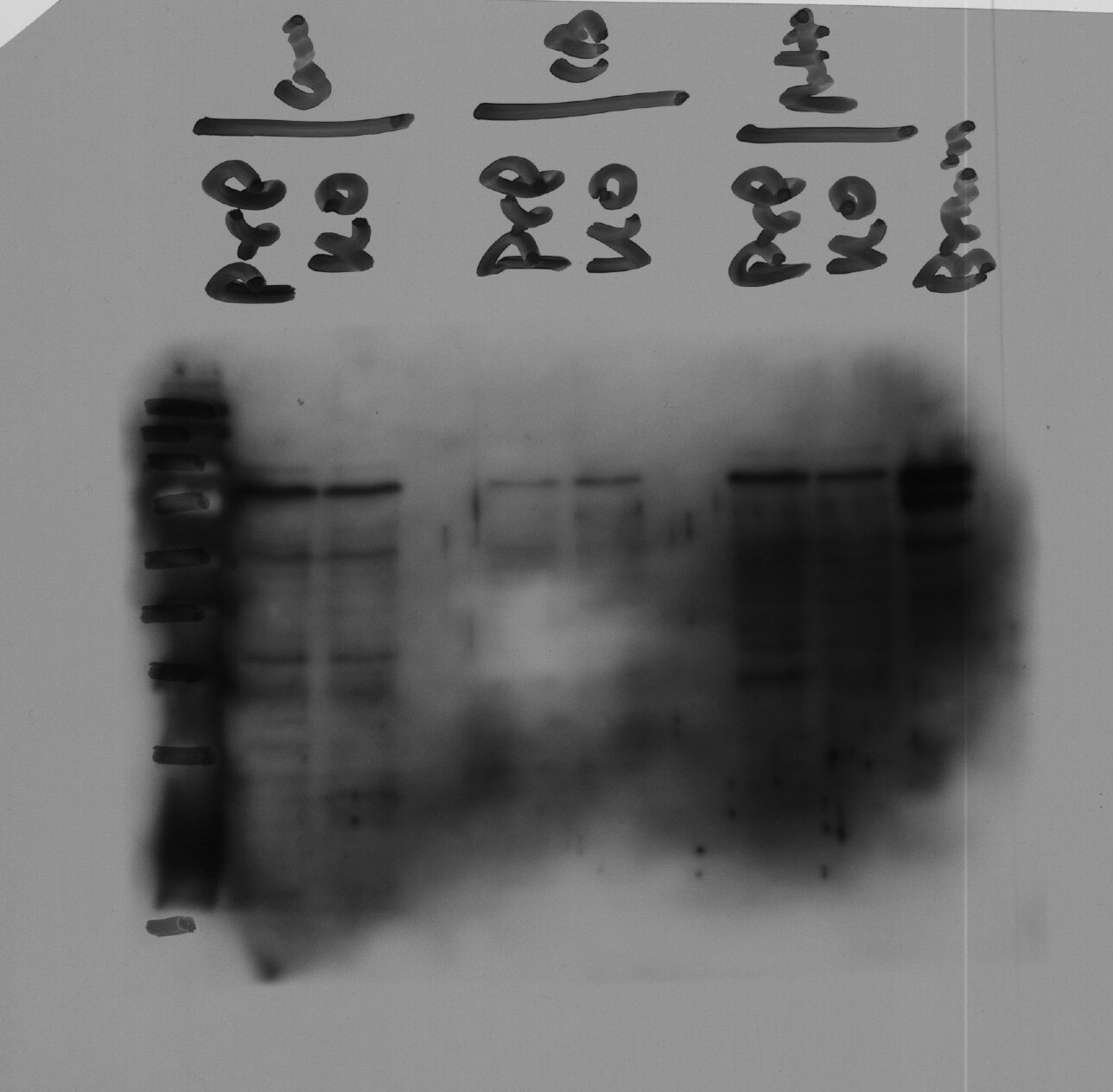
**Figure 7B-**Order of probing: 1) Tf

78

Lane: 1 2 3 4

**Gapdh**


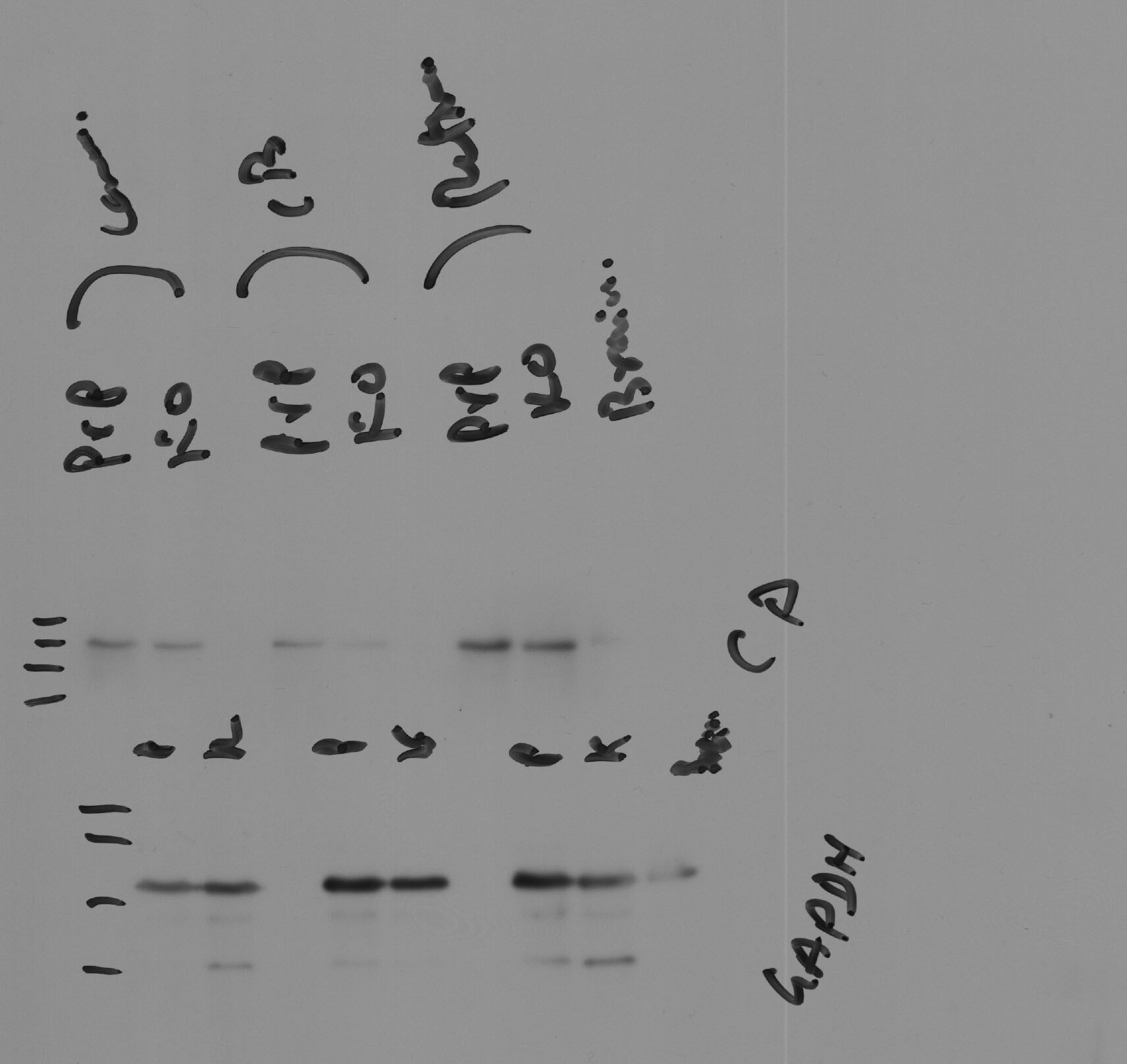
 **Figure 7B-**Order of probing: 1) Tf, 2) Cp 3) Gapdh

36

Lane: 1 2 3 4

**TfR**


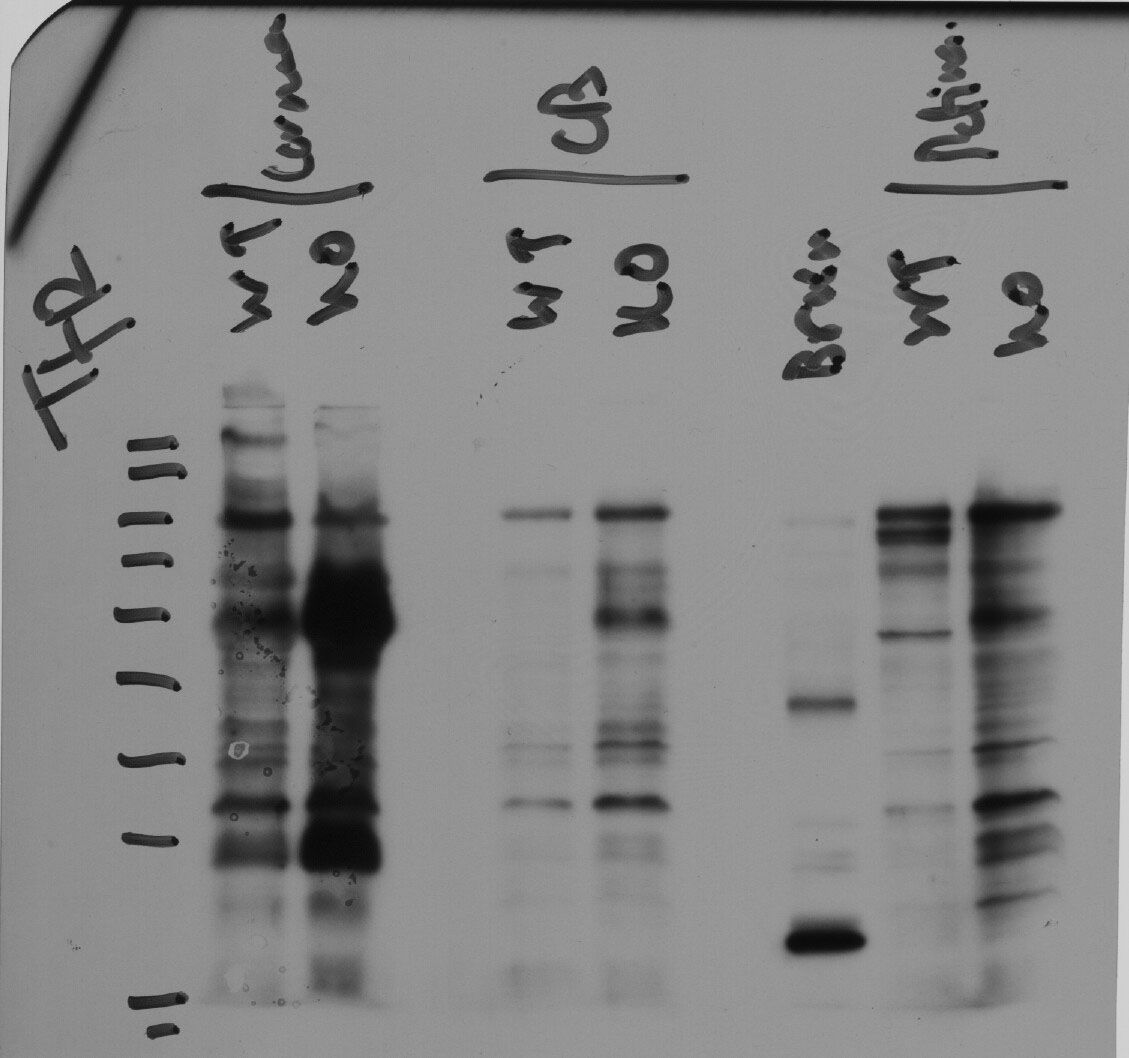
 **Figure 7B-**Order of probing: 1) PrP (used in Figure 6B), 2)

94

Lane: 1 2 3 4

Ferritin 3) TfR

**Ferritin**

**
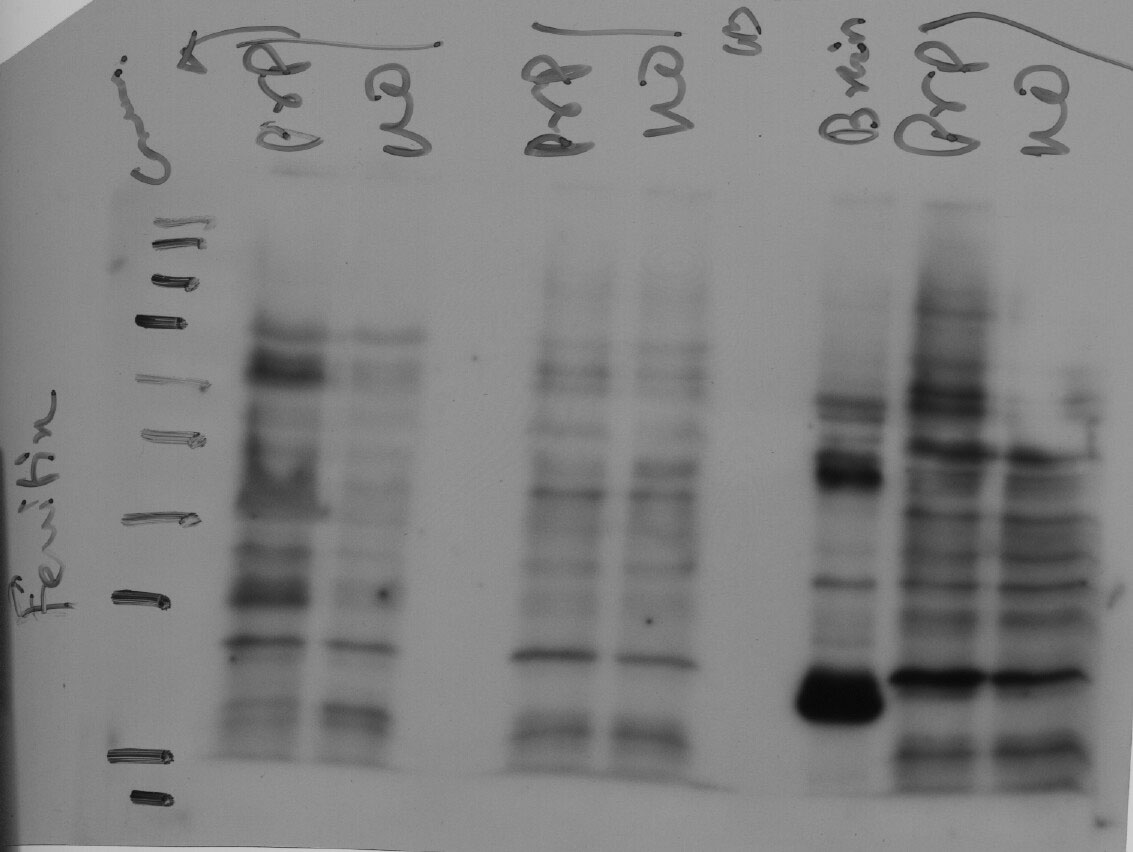
 Figure 7B-**Order of probing: 1) PrP (used in Figure 6B), 2)

20

Lane: 1 2 3 4

Ferritin 3) TfR

**β-actin**

**
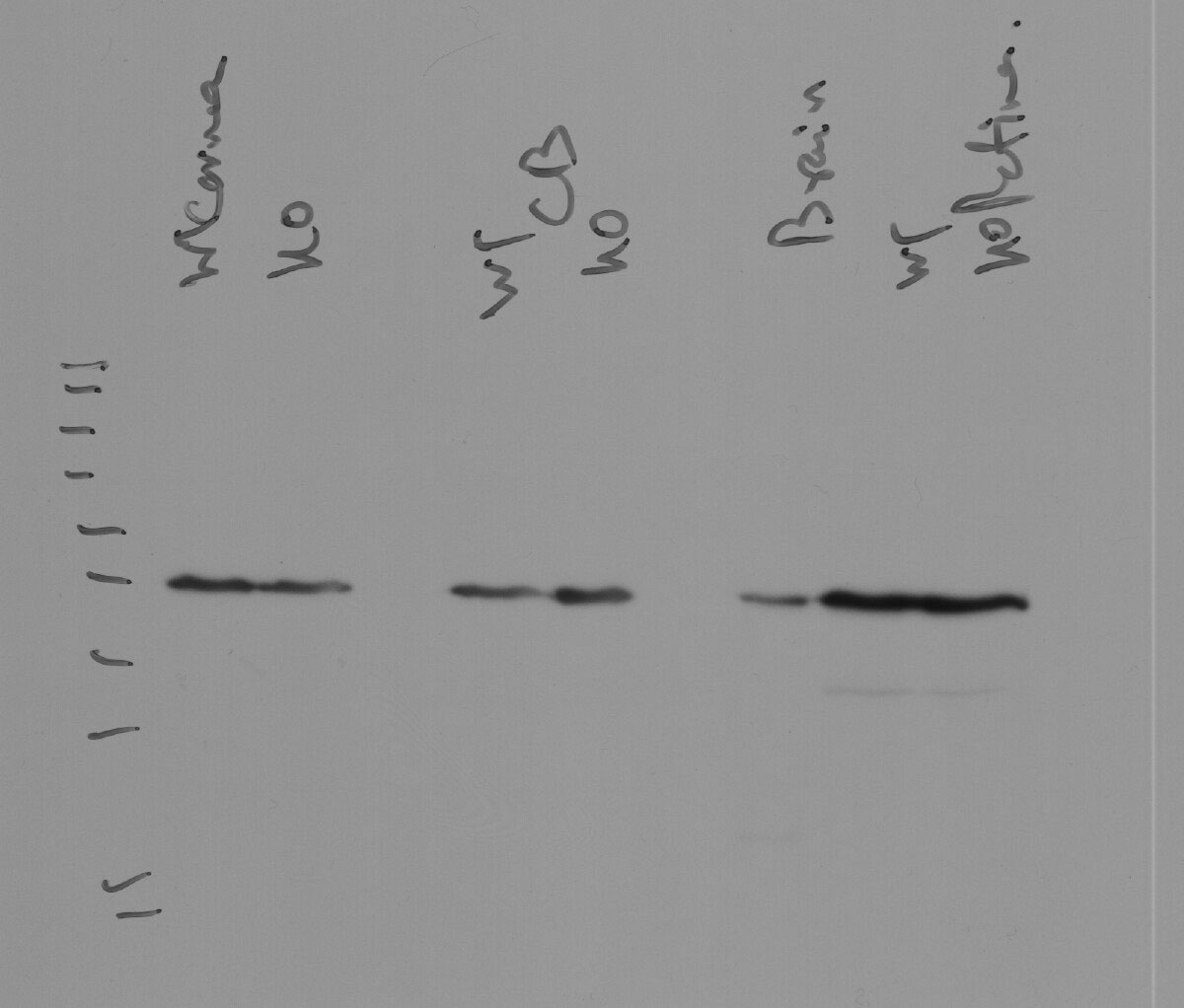
 Figure 7B-**Order of probing: 1) PrP (used in Figure 6B), 2)

42

Lane: 1 2 3 4

Ferritin, 3) TfR 4) β actin

**Figure 8**

**Figure 8A-The membranes were reprobed**

**PrP**


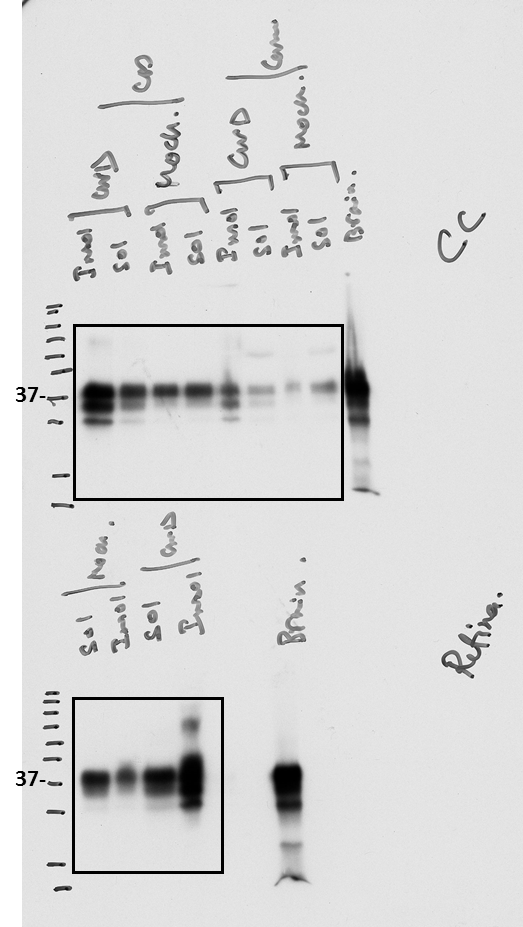
 **Figure 8A-**Order of probing: 1)PrP(SAF32)

Lane: 9 10 11 12

**Gapdh**


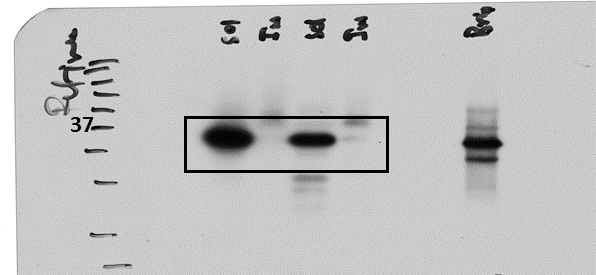
 **Figure 8A-**Order of probing: 1) PrP(SAF32),

Lane: 9 10 11 12

**PrP**


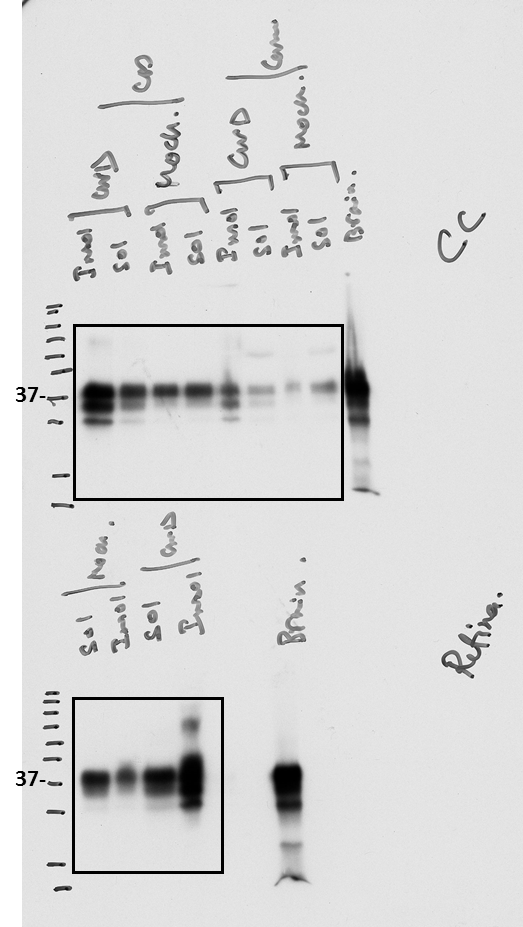
 **Figure 8A-**Order of probing: 1)PrP(SAF32)

Lane: 8 7 6 5 4 3 2 1

**Gapdh**


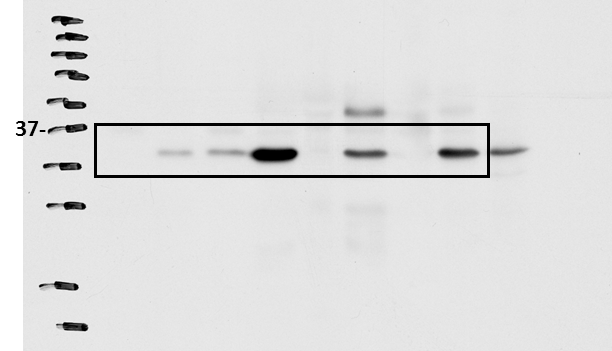
 **Figure 8A-**Order of probing: 1)PrP(SAF32),

Lane: 8 7 6 5 4 3 2 1

2) Gapdh
